# SreC‐dependent adaption to host iron environments regulates the transition of trophic stages and developmental processes of *Curvularia lunata*

**DOI:** 10.1111/mpp.13444

**Published:** 2024-03-13

**Authors:** Jiaying Sun, Jiamei Zhao, Miaomiao Liu, Jiayang Li, Jie Cheng, Wenling Li, Mingyue Yuan, Shuqin Xiao, Chunsheng Xue

**Affiliations:** ^1^ College of Plant Protection Shenyang Agriculture University Shenyang China; ^2^ Section of Microbial Ecology, Department of Biology Lund University Lund Sweden

**Keywords:** GATA transcription factor SreC, hemibiotrophic fungi, host iron environment, iron assimilation pathways, trophic stage, virulence

## Abstract

Plant pathogens are challenged by host‐derived iron starvation or excess during infection, but the mechanism of plant pathogens rapidly adapting to the dynamic host iron environments to assimilate iron for invasion and colonization remains largely unexplored. Here, we found that the GATA transcription factor SreC in *Curvularia lunata* is required for virulence and adaption to the host iron excess environment. SreC directly binds to the ATGWGATAW element in an iron‐dependent manner to regulate the switch between different iron assimilation pathways, conferring adaption to host iron environments in different trophic stages of *C. lunata*. SreC also regulates the transition of trophic stages and developmental processes in *C. lunata*. SreC‐dependent adaption to host iron environments is essential to the infectious growth and survival of *C. lunata*. We also demonstrate that CgSreA (a SreC orthologue) plays a similar role in *Colletotrichum graminicola*. We conclude that Sre mediates adaption to the host iron environment during infection, and the function is conserved in hemibiotrophic fungi.

## Graphical abstract

Transcription factor SreC regulates iron assimilation pathways switch in adaption to host iron environment in different trophic stages during *Curvularia lunata* infection, the function is conserved in hemibiotrophic fungi.

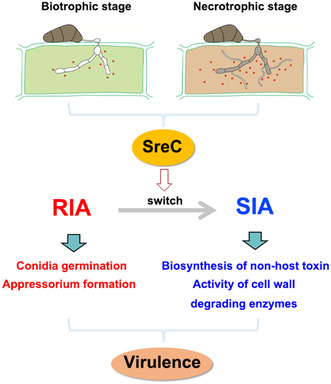

## 1 INTRODUCTION

Iron (Fe) is an essential micronutrient for almost all organisms, including plants and their pathogens. Sufficient iron absorption is crucial for pathogens to colonize and develop during infection (Singh et al., 2016; Wang, Zhang, et al., 2022; Xing et al., 2021). Fungi acquire iron from their hosts by employing two high‐affinity iron uptake systems: reductive iron assimilation (RIA) and siderophore‐mediated iron assimilation (SIA) (Albarouki & Deising, 2013; Birch & Ruddat, 2005; Haas et al., 2008). Deletion of gene *Fer1* (iron multicopper oxidase) and *Fer2* (iron permease) in the RIA pathway results in development defects and virulence reduction of *Ustilago maydis* and *Microbotryum violaceum* (Eichhorn et al., 2006; Liu et al., 2020; Schrettl et al., 2010). The non‐ribosomal peptide synthases (Nps) related to siderophore biosynthesis in the SIA pathway are required for full virulence, vegetative growth, asexual sporulation and/or oxidative stress tolerance under iron‐limiting conditions in *Cochliobolus heterostrophus*, *Fusarium graminearum*, *Cochliobolus miyabeanus*, *Alternaria brassicicola* and *C. lunata* (Condon et al., 2014; Greenshields et al., 2007; Lu et al., 2020; Oide et al., 2006; Voisard et al., 1993).

To prevent pathogen infection, host plants redistribute iron at the cellular level to initiate iron immunity (Fu et al., 2022; Ganz & Nemeth, 2015; Kehl‐Fie & Skaar, 2010; Soares & Weiss, 2015; Weinberg & Miklossy, 2008). Some host plants employ iron‐withholding strategies, such as iron‐sequestering ferritin and defensins, to reduce pathogen proliferation (Expert et al., 1996; Franza & Expert, 2013; Lemanceau et al., 2009). On the other hand, some plants can locally accumulate iron to generate highly toxic hydroxyl radicals to prevent pathogen infection (Aznar et al., 2015; Greenshields et al., 2007; Verbon et al., 2017). For example, maize, under an adequate iron nutritional status, can recruit free iron to the infection site, which induces reactive oxygen species (ROS) accumulation to suppress infection by the fungus *Colletotrichum graminicola* (Ye et al., 2014). Notably, hemibiotrophic fungi such as *C. graminicola* employ the RIA and SIA pathways to assimilate iron in the biotrophic and necrotrophic stages, respectively (Albarouki et al., 2014). The RIA pathway in *C. graminicola* is involved in regulating the penetration ability of appressoria in the biotrophic stage, while the SIA pathway is involved in hyphal growth and virulence in the necrotrophic stage (Albarouki & Deising, 2013). These findings suggest that hemibiotrophic fungi switch between high‐affinity iron assimilation pathways to adapt to drastic changes of the host iron environment to ensure full virulence during infection; however, the key mechanism of the switch between iron assimilation pathways is still unknown.

Alteration of the host iron status can interfere with iron intracellular homeostasis of pathogens during infection (Expert et al., 1996; Verbon et al., 2017). Therefore, pathogens have developed sophisticated strategies to control iron acquisition, consumption, and storage (Canessa & Larrondo, 2013; Gu et al., 2022; John et al., 2021; Sun et al., 2023). In fungi, the GATA‐type transcription factor Sre (siderophore regulator) and its orthologues are known for their involvement in maintaining appropriate iron levels to ensure optimal cellular function and avoid iron toxicity (Chen et al., 2013; López‐Berges et al., 2012; Wang et al., 2019; Zhang et al., 2013). Under iron‐replete conditions, Sre suppresses the transcription of genes in both the RIA and SIA pathways (Chao et al., 2008; Voisard et al., 1993). Under iron‐deficient conditions, the transcriptional inhibition of Sre is eliminated, so that phytopathogenic fungi can rapidly take up iron from the hosts (Schrettl et al., 2010; Zhang et al., 2013). In *Ustilago maydis*, the transcription factor Urbs1 (*Ustilago* kinase B‐related 1) specifically binds to the siderophore biosynthesis gene *Sid1* (L‐ornithine N^5^‐oxygenase) promoter region (An, Mei, et al., 1997; An, Zhao, et al., 1997). In *Alternaria alternata*, SreA directly binds the *Nps6* promoter to inhibit its transcription and consequently interrupt siderophore biosynthesis (Chung et al., 2020). These findings suggest that Sre may be a key factor in regulating the switch between iron assimilation pathways from RIA to SIA; however, the key mechanism of transcriptional regulation remains to be documented.

*Curvularia lunata* is a hemibiotrophic pathogenic fungus that causes leaf spot on maize, rice, sorghum and barley (Lanisnik Rizner & Wheeler, 2003). Curvularia leaf spot caused by *C. lunata* is one of the common foliar fungal diseases of maize, is widely distributed worldwide and is associated with a severe yield loss, especially in warm and humid maize production regions (Henrickson & Koehler, 2020; Macri & Di Lenna, 1974; Wang, Lu, et al., 2022). Upon landing on the maize leaf surface, *C. lunata* conidia germinate and differentiate into appressoria, which breach the host cuticle and cell wall, then form invasive primary hyphae inside plant cells. The primary hyphae of *C. lunata* are entirely confined to the initially infected epidermal cells throughout the biotrophic phase. After that, the fungus switches to a necrotrophic phase associated with thin, melaninized, and fast‐growing secondary hyphae that grow intra‐ and intercellulary to kill and destroy host tissues via hydrolytic enzymes and the nonhost‐selective toxin methyl 5‐(hydroxy‐methyl) furan‐2‐carboxylate (M5HF2C) (Liu et al., 2009). Transition from the asymptomatic biotrophic phase, characterized by intercellular thick primary hyphae, to the destructive necrotrophic phase, characterized by thin filamentous secondary hyphae, is referred to as the biotrophy–necrotrophy switch of hemibiotrophic fungi (Lee & Rose, 2010). Our previous study found that the transition from the biotrophic phase to necrotrophic phase at 24 h post‐inoculation (hpi) matches the switch of iron assimilation pathways, which permits full virulence of *C. lunata* (Lu et al., 2020). Here, we show that *C. lunata* is exposed to drastic changes of the host iron environments during infection. To adapt to these iron environments, the transcription factor SreC regulates the iron assimilation pathways by directly binding to the ATGWGATAW element in the gene promoters in an iron‐dependent manner. Furthermore, we provide pieces of evidence that SreC mediates the switch of iron assimilation pathways in *C. lunata*. Finally, we found that the SreC is vital to the transition of trophic stages and developmental processes during *C. lunata* infection. We also confirm that the *C. graminicola CgSreA* (orthologue) has similar functions in adaption to the host iron environment for full virulence, suggesting the conserved role of Sre in hemibiotrophic fungi.

## 2 RESULTS

### 2.1 SreC is a key regulator in *C. lunata* to adapt to the host iron‐excess environment

**FIGURE 1:**
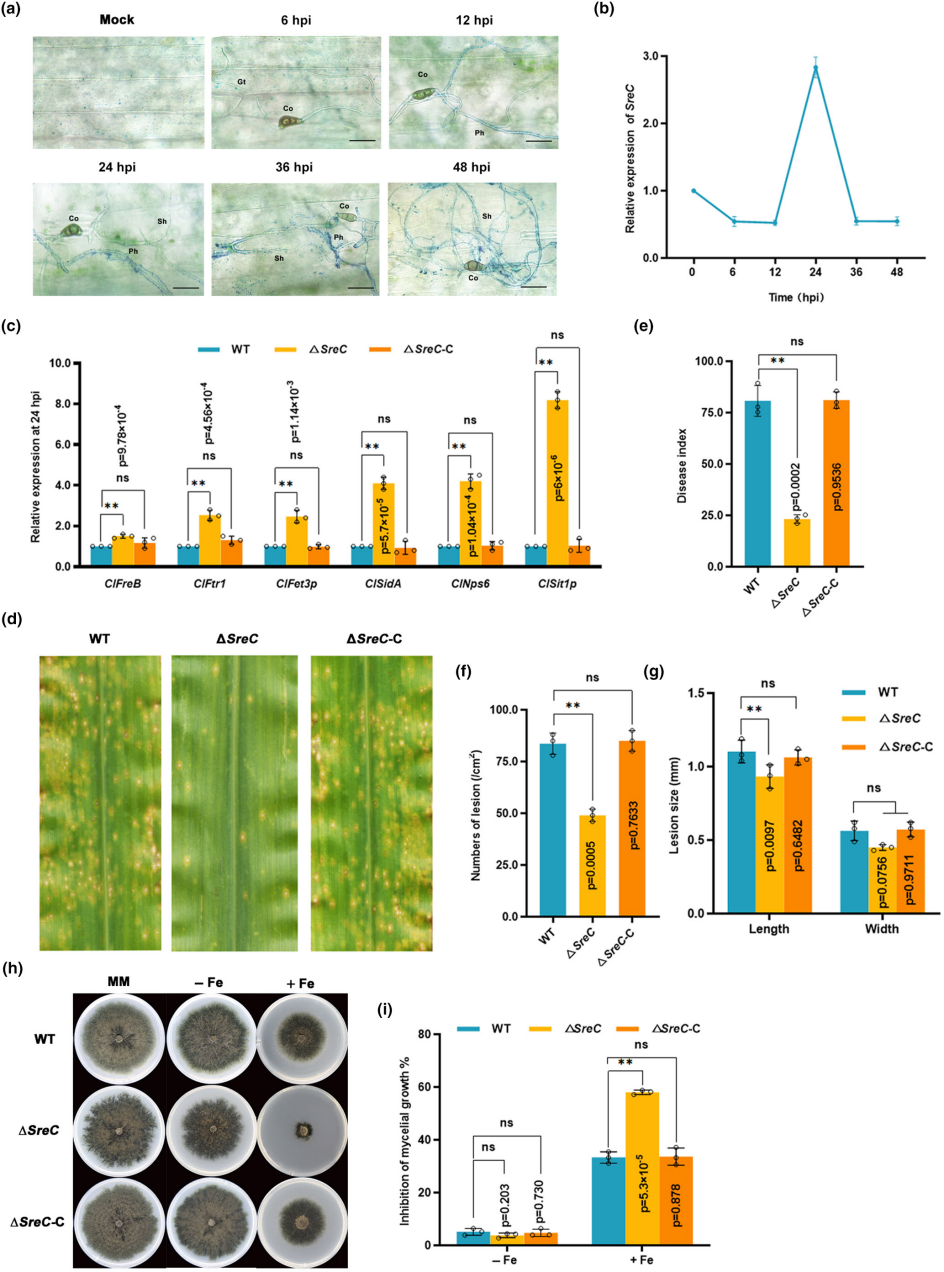
SreC regulates the adaption to the host iron‐excess environment. (a) Prussian blue staining of infected leaf epidermal cells during *Curvularia lunata* infection. The barley leaves were inoculated with *C. lunata* conidia at a concentration of 10^5^ conidia/mL. Co, conidium; Gt, germination tube; Ph, primary infection hypha; Sh, secondary hypha. Bar, 25 μm. (b) The expression profiles of *SreC* in infected maize leaves by *C. lunata*. The maize leaves were inoculated with *C. lunata* conidia at a concentration of 10^6^ conidia/mL. The leaves were sampled at the indicated time points for reverse transcription‐quantitative PCR assays. The expression of *SreC* at 0 h post‐inoculation (hpi) was set as 1. *C. lunata ClActin* was used as the reference gene. (c) The expression of iron assimilation pathways genes in wild‐type (WT), Δ*SreC*, and Δ*SreC*‐C at 24 hpi. Others were as in (b). (d) Disease symptom of maize leaves infected with WT, Δ*SreC*, and Δ*SreC*‐C at 7 days post‐inoculation (dpi). Conidial suspensions (10^6^ conidia/mL in 0.02% Tween 20) were sprayed onto the leaf surfaces of maize at eighth‐leaf stage. (e) Disease index of maize leaves infected with WT, Δ*SreC*, and Δ*SreC*‐C. (f) The number of lesions per unit area of lesions infected with WT, Δ*SreC*, and Δ*SreC*‐C. (g) Length and width of the chlorosis and necrosis symptoms associated with individual lesions infected with WT, Δ*SreC*, and Δ*SreC*‐C. Others were as in (d). (h) Sensitivity of WT, Δ*SreC*, and Δ*SreC*‐C to iron stress. A 5 mm mycelial plug of each strain was inoculated on minimal medium (MM) with 50 μM bathophenanthroline disulfonate (BPS) (−Fe) or 1 mM FeCl_3_ (+Fe), then incubated at 28°C for 7 days. (i) Mycelial growth inhibition of WT, Δ*SreC*, and Δ*SreC*‐C to iron stress. Mycelial growth inhibition of each treatment was calculated at 7 dpi. Values are means ± *SD* (*n* = 3 biological replicates). An asterisk indicates significant differences based on unpaired two‐tailed Student's *t* test with the *p* values marked (*p < 0.05, **p < 0.01, ns, not significant).

Plants redistribute iron at the cellular level to suppress fungal infection (Liu et al., 2007; Ye et al., 2014). To investigate the change of iron status in host plant cells during *C. lunata* infection, we characterized the Fe^3+^ localization in barley leaves infected by *C. lunata* at the cellular level using Prussian blue staining. As shown in Figure 1a, abundant iron significantly accumulated in barley leaf cells from 24 to 48 hpi. This result indicated that upon infection by *C. lunata*, iron was redistributed in plant cells. Transcription factor Sre orthologues have been reported to be responsible for iron resistance in fungi (Chung et al., 2020; Oberegger et al., 2001; Schrettl et al., 2010). From the reverse transcription‐quantitative PCR (RT‐qPCR) assay, we found that expression of the transcription factor *SreC* (GenBank accession KJ556848.1) was induced by local iron accumulation in maize leaf cells (Figure 1b, 24 hpi). RT‐qPCR assays also confirmed that the expression of key iron uptake genes in RIA and SIA pathways were significantly up‐regulated in Δ*SreC* at 24 hpi (Figure 1c). Deletion of *SreC* resulted in significant virulence reduction (Figure 1d,e), and the lesion numbers and sizes on leaves showed significantly attenuated symptoms at 7 dpi (Figure 1d,f,g). The Δ*SreC* exhibited significantly increased sensitivity to iron, when iron is in excess (Figure 1h,i). Collectively, these results indicated that SreC negatively regulated the expression of iron uptake genes, thus conferring adaption to the iron‐excess environment during *C. lunata* infection.

### 2.2 SreC regulates the RIA and SIA pathways in an iron‐dependent manner

**FIGURE 2:**
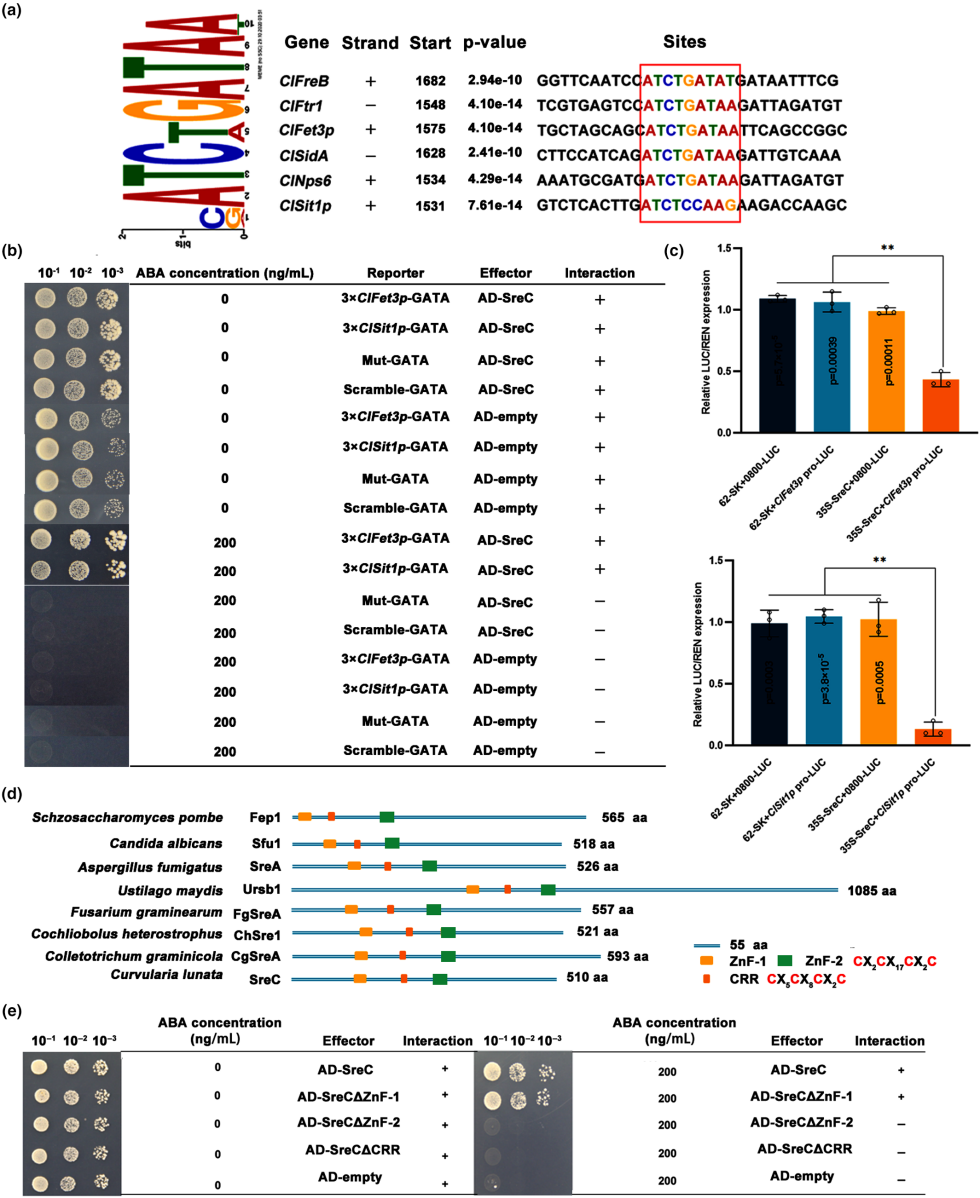
SreC transcriptionally regulates the switch of reductive iron assimilation (RIA) to the siderophore‐mediated iron assimilation (SIA) pathway. (a) Identification of SreC binding motifs using the multiple EM for motif elicitation (MEME) program. The identified binding *cis*‐elements of iron assimilation pathways genes are indicated in the red square. (b) Physical interaction of SreC with the promoters of *ClFet3p* and *ClSit1p* in yeast one‐hybrid (Y1H) system. Aureobasidin A (ABA) was added to the medium to inhibit the self‐activation of the *ClFet3p* and *ClSit1p* promoters. The negative control was mutant and scrambled GATA motif with AD‐SreC. The concentration of ABA added to synthetic dropout (SD) medium lacking leucine (L) (SD−L) was 200 ng/mL. (c) Dual‐luciferase (LUC) assay in *Nicotiana benthamiana* cells showed binding of SreC with the promoters of *ClFet3p* and *ClSit1p* genes. All luciferase genes are expressed relative to the control (value set at 1). Values are means ± *SD* (*n* = 3 biological replicates). An asterisk indicates significant differences based on unpaired two‐tailed Student's *t* test with the *p* values marked (*p < 0.05, **p < 0.01, ns, not significant). (d) Schematic view of two zinc finger (ZnF) domains and a cysteine‐rich central (CRR) domain of SreC. (e) The C‐terminal zinc finger (ZnF‐2) and CRR domains of SreC were required for its interaction with the promoters of iron assimilation pathways genes in the Y1H system. Others were as in (b).

We next aimed to elucidate the regulatory mechanisms of SreC on RIA and SIA pathways in iron‐excess environments. The multiple EM for motif elicitation (MEME) program indicated that *ClFreB*, *ClFtr1*, and *ClFet3p* genes in the RIA pathway, and *ClSidA*, *ClNps6*, and *ClSit1p* genes in the SIA pathway harboured the conserved SreC‐binding *cis*‐element ATGWGATAW (where W = A/T) (Figure 2a). The binding of SreC with the ATGWGATAW element in the promoters of these genes was further confirmed via yeast one‐hybrid (Y1H) assays (Figures S3b and 2b). A dual‐luciferase reporter assay further confirmed that SreC bound to the promoters of *ClFet3p* and *ClSit1p* (Figure 2c). *Nicotiana benthamiana* leaves co‐transformed with 35S‐SreC and Pro‐LUC displayed a significant down‐regulation of luminescence due to expression of the luciferase gene, and the relative LUC/REN expression was 0.4‐ and 0.15‐fold lower than those of the leaves with control vectors (Figure 2c). These results indicated that SreC transcriptionally repressed the RIA and SIA pathways in response to iron‐excess environments.

**FIGURE 3:**
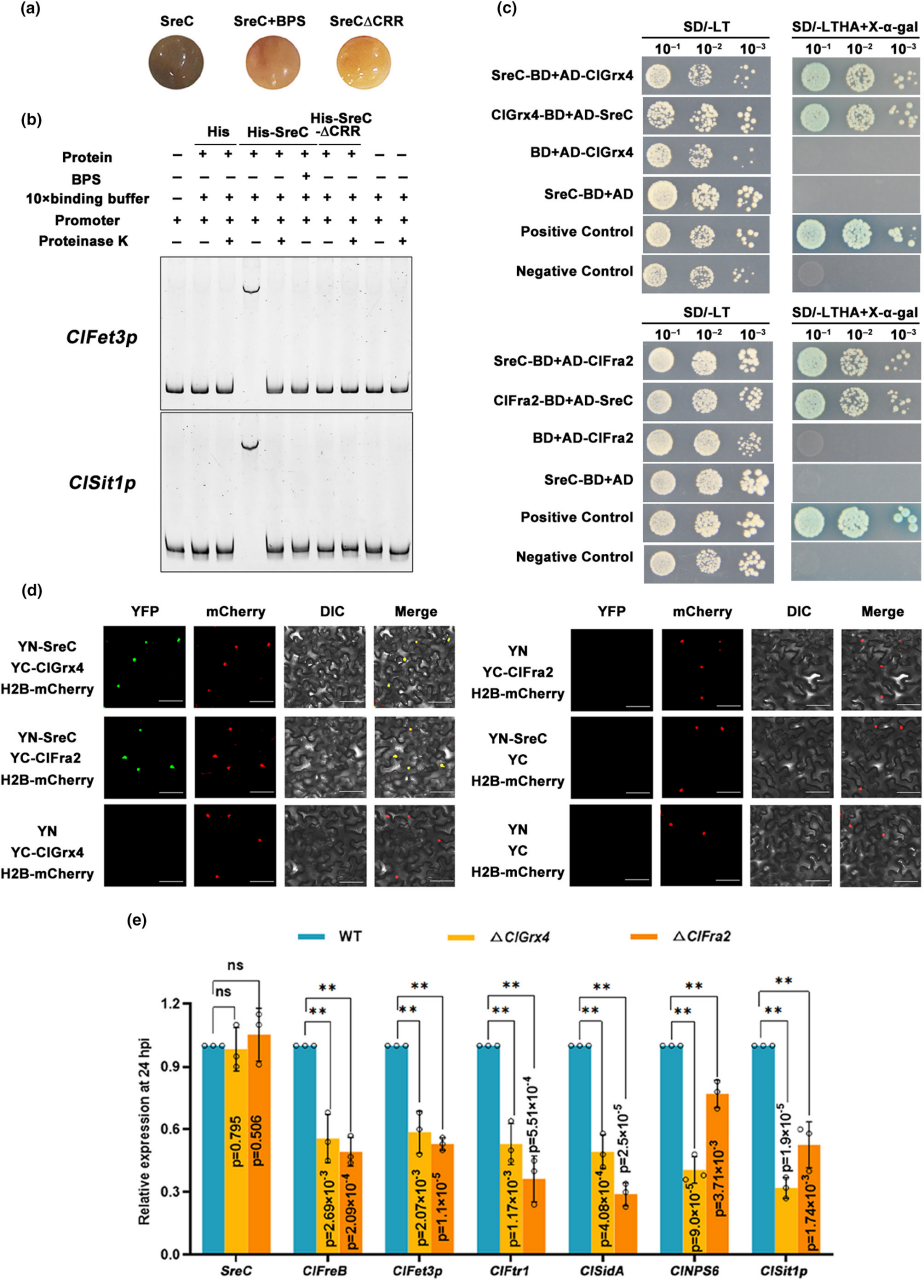
SreC transcriptionally regulates reductive iron assimilation (RIA) to siderophore‐mediated iron assimilation (SIA) pathway in an iron‐dependent manner. (a) Iron binding of SreC was dependent on the cysteine‐rich central (CRR) domain. *Escherichia coli* cells with pET28‐*SreC* were cultured on media containing 1 mM IPTG, with 1 mM FeCl_3_ (+Fe) and 50 μM bathophenanthroline disulfonate (BPS) (−Fe), at 37°C for 4 h. Cells were centrifuged and cell pellets were photographed. (b) Iron was required for SreC binding to promoters of *ClFet3p* and *ClSit1p* as visualized by electrophoretic mobility shift assay (EMSA). The DNA probe was amplified using the *ClFet3p* and *ClSit1p* promoter region containing the ATGWGATAW element. His‐SreC and His‐SreC‐∆CRR were produced heterologously in *E. coli* and purified. The DNA probe was incubated with purified His‐SreC, His‐SreC‐∆CRR, and His with or without proteinase K for 20 min at 25°C. (c) SreC interacted with ClGrx4 and ClFra2 in yeast two‐hybrid assay. Serial dilutions of the yeast cells were plated on synthetic dropout (SD) medium lacking leucine (L), tryptophan (T), histidine (H), and adenine (A) (SD−LTHA). The yeast strain containing pGBKT7‐53 and pGADT7 was used as a positive control, whereas that containing pGBKT7‐Lam and pGADT7 was used as a negative control. (d) The interaction of SreC and ClGrx4 or ClFra2 in the nucleus as visualized by bimolecular fluorescence complementation. A pair of constructs SreC‐CYFP+NYFP, and another pair of constructs ClGrx4/ClFra2‐NYFP+CYFP were used as negative controls. Yellow fluorescent protein (YFP) signals were observed using confocal microscopy. The nuclear localization was confirmed by simultaneous nuclear labelling with H2B‐mCherry. DIC, differential interference contrast microscopy. (e) The expression of iron assimilation pathways genes in wild‐type (WT), Δ*ClGrx4*, and Δ*ClFra2* at 24 h post‐inoculation (hpi). The maize leaves were inoculated with *Curvularia lunata* conidia at a concentration of 10^6^ conidia/mL. The leaves were sampled at the indicated time for reverse transcription‐quantitative PCR assays. *C. lunata ClActin* was used as the reference gene. Values are means ± *SD* (*n* = 3 biological replicates). An asterisk indicates significant differences based on unpaired two‐tailed Student's *t* test with the *p* values marked (**p < 0.01, ns, not significant).

Sre contains two zinc finger (GATA) domains, of which the C‐terminal zinc finger is essential for DNA binding and the N‐terminal zinc finger enhances DNA binding affinity, as well as a cysteine‐rich central (CRR) domain for sensing iron ions (del Dedo et al., 2015; Pelletier et al., 2005; Schrettl et al., 2008). The amino acid sequences of three domains in SreC are highly similar to its orthologues (Figure S1). SreC contains two zinc finger motifs (denoted ZnF‐1 and ZnF‐2) and a conserved 32 amino acid segment containing four invariant cysteine residues (denoted CRR) that is located between the two zinc finger motifs (Figure 2d). To verify the role of domains in SreC, we constructed a SreC domain deletion vector by the point mutation technique. The results showed that the C‐terminal zinc finger was essential for SreC to directly bind to the promoters of genes in the RIA and SIA pathways and suppress their transcription (Figure 2e). In addition, yeast strains containing the ATGWGATAW element and the SreC‐∆CRR plasmids failed to grow on selective medium supplemented with aureobasidin A (ABA), which inhibits Y1H bait self‐activation (Figure 2e). During the Ni‐affinity purification of recombinant SreC in *Escherichia coli*, we observed that the eluted protein was reddish brown. Interestingly, the introduction of the C207A/C213A substitutions in the cysteine‐rich region of SreC fully abrogated the reddish‐brown colour of the harbouring cells, which is consistent with bacterial colonies producing SreC when grown in medium containing bathophenanthroline disulfonate (BPS) (Figure 3a). Amino acid substitutions in the CRR domain in His‐SreC also resulted in the loss of binding ability to *ClFet3p* and *ClSit1p* promoters (Figure 3b). These results indicated that iron was required for SreC transcriptional repression of the RIA and SIA pathways.

The Grx4–Fra2 heterodimer constitutively binds to Sre, and iron is transferred from Sre to Grx4–Fra2 and derepresses iron assimilation genes under iron‐deprivation conditions (del Dedo et al., 2015; Sun et al., 2023). In this work, we confirmed the interaction between ClGrx4 and ClFra2 (Figure S4a). Yeast two‐hybrid (Y2H) and bimolecular fluorescence complementation (BiFC) assays revealed that SreC interacted with ClGrx4 and ClFra2 in the nucleus (Figure 3c,d). We also found that Δ*ClGrx4* and Δ*ClFra2* exhibited significantly increased sensitivity to iron excess (Figure S4b,c) and the expression of genes in RIA and SIA pathways were significantly suppressed at 24 hpi (Figure 3e). When considered together, these results suggested that the ClGrx4–ClFra2 heterodimer mediated SreC transcriptional regulation of the RIA and SIA pathways.

### 2.3 SreC is involved in the switch of iron assimilation pathways in *C. lunata* during infection

**FIGURE 4:**
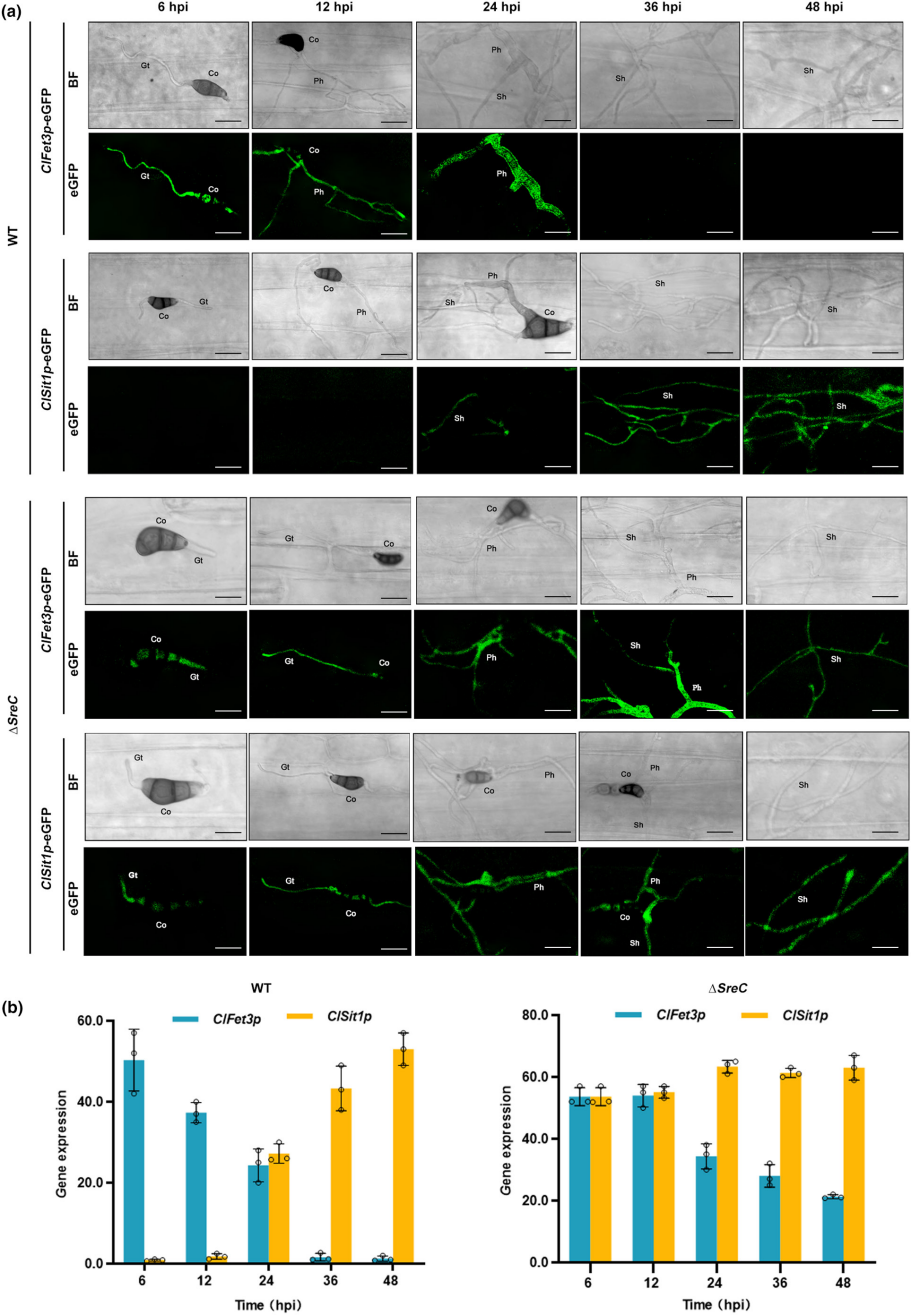
SreC is involved in the switch between iron assimilation pathways during infection in *Curvularia lunata*. (a) Trophic stage‐specific expression of *ClFet3p* (in the reductive iron assimilation pathway) and *ClSit1p* (in the siderophore‐mediated iron assimilation pathway) during wild‐type (WT) and Δ*SreC* infection. The expression of *ClFet3p*‐eGFP and *ClSit1p*‐eGFP fusion constructs were assessed in WT and Δ*SreC* strains. Conidial suspensions (10^6^ conidia/mL in 0.02% Tween 20) were sprayed onto the leaf surfaces of 7‐day‐old barley plants. Co, conidium; Gt, germination tube; Ph, primary infection hypha; Sh, secondary infection hypha. Bar, 25 μm. hpi, hours post‐inoculation. (b) Expression profiles of *ClFet3p* and *ClSit1p* during WT and Δ*SreC* infections. The maize leaves were inoculated with WT and Δ*SreC* conidia at a concentration of 10^6^ conidia/mL. The leaves were sampled at indicated time for reverse transcription‐quantitative PCR assays. *C. lunata ClActin* was used as the reference gene. Values are means ± *SD* (*n* = 3 biological replicates).

Our previous work revealed that *C. lunata* absorbed iron with the RIA and SIA pathways in the biotrophic and necrotrophic stages, respectively (Lu et al., 2020). Here, we performed infection with *ClFet3p*‐eGFP and *ClSit1p‐*eGFP strains, which are marker genes in the RIA and SIA pathways, respectively. In both strains, eGFP fluorescence was stronger under iron‐limited conditions and weaker under iron‐sufficient conditions (Figure S5c,d). *ClFet3p*‐eGFP showed strong fluorescence in the biotrophic stage (Figure 4a, 6–24 hpi), then fluorescence of secondary hyphae rapidly disappeared in the necrotrophic stage (Figure 4a, 24–48 hpi). In contrast, *ClSit1p*‐eGFP displayed no fluorescence in primary hyphae in the biotrophic stage (Figure 4a, 6–24 hpi), but fluorescence occurred in secondary hyphae in the necrotrophic stage (Figure 4a, 24–48 hpi). To test whether *SreC* regulated the switch between iron assimilation pathways in *C. lunata*, we generated *ClFet3p‐*eGFP and *ClSit1p‐*eGFP in the ∆*SreC* background. We found that both ∆*SreC‐ClFet3p*‐eGFP and ∆*SreC‐ClSit1p*‐eGFP strains showed strong fluorescence in biotrophic and necrotrophic stages (Figure 4a, 6–48 hpi). Furthermore, expression of *ClFet3p* and *ClSit1p* showed continuously high levels, with no obvious difference between biotrophic and necrotrophic stages (Figure 4a). The expression profiles of these two genes were consistent with the observations of eGFP fluorescence in wild‐type (WT) CX‐3 and ∆*SreC* (Figure 4b). These results indicated that the absence of *SreC* led to an interruption of the switch from the RIA to the SIA pathway. Here, we found that the switch between RIA and SIA pathways was also abnormal in Δ*ClGrx4* and Δ*ClFra2* (Figure S4d). When considered together, these results suggested that SreC was dependent on the ClGrx4–ClFra2 heterodimer to sense drastic changes in the host iron environment, subsequently regulating the switch of iron assimilation pathways in *C. lunata*.

### 2.4 SreC is essential for transition of trophic stages and developmental processes of *C. lunata* during infection

**FIGURE 5:**
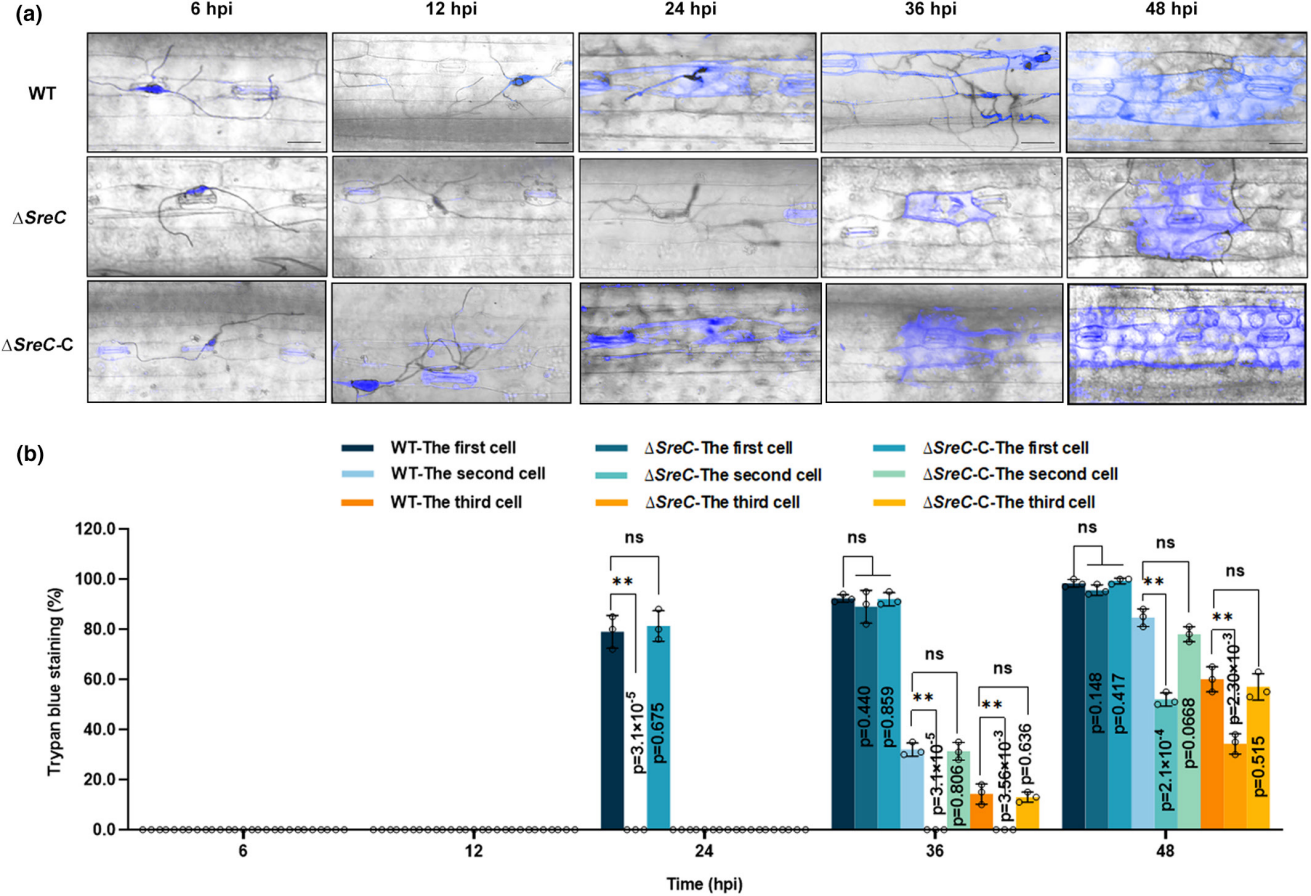
SreC is essential for the transition of trophic stages by *Curvularia lunata* during infection. (a) Trypan blue staining of infected leaf epidermal cells during wild‐type (WT), Δ*SreC*, and Δ*SreC*‐C infection. The barley leaves were inoculated with WT, Δ*SreC*, and Δ*SreC*‐C conidia at a concentration of 10^5^ conidia/mL. Bar, 25 μm. hpi, hours post‐inoculation. (b) Percentage of infected leaf cells stained by trypan blue during WT, Δ*SreC*, and Δ*SreC*‐C infection. Values are means ± *SD* (*n* = 3 biological replicates). An asterisk indicates significant differences based on unpaired two‐tailed Student's *t* test with the *p* values marked (**p < 0.01, ns, not significant).

The viability of infected host cells was assessed using trypan blue staining. We observed that the first plant cells infected by WT CX‐3 and Δ*SreC*‐C at infection sites were stained at 24 hpi (Figure 5a,b). At 36 hpi, secondary plant cells infected by WT CX‐3 and Δ*SreC*‐C at infection sites lost viability (Figure 5a,b). In contrast, no cells were found to be stained with trypan blue at the infection site by Δ*SreC* at 24 hpi (Figure 5a,b) and the first infected plant cells at infection sites were stained at 36 hpi (Figure 5a,b). At 48 hpi, secondary plant cells infected by Δ*SreC* at infection sites lost viability (Figure 5a,b). These results indicated that the transition from biotrophic to necrotrophic stages in Δ*SreC* was significantly delayed.

**FIGURE 6:**
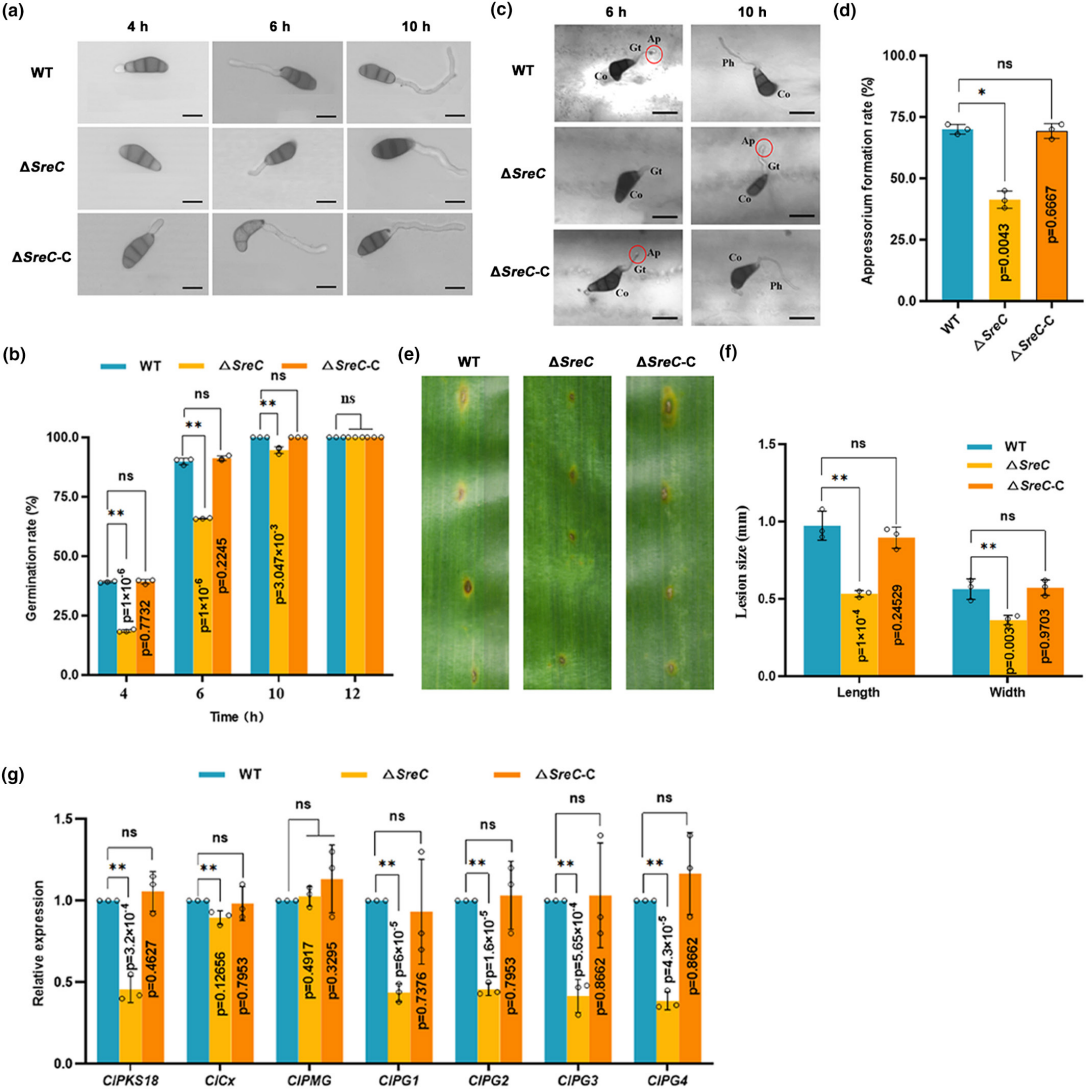
SreC is essential for developmental processes of *Curvularia lunata* during infection. (a) Conidial germination process of wild type (WT), Δ*SreC*, and Δ*SreC*‐C. Bar, 25 μm. (b) Conidial germination rates of WT, Δ*SreC*, and Δ*SreC*‐C. (c) Appressorial differentiation of WT, Δ*SreC*, and Δ*SreC*‐C at 6–10 h post‐inoculation (hpi). The maize leaves were inoculated with WT, Δ*SreC*, and Δ*SreC*‐C conidia at a concentration of 10^5^ conidia/mL. Co, conidia; Ap, appressorium; Gt, germination tube; Ph, primary hypha. Bar, 10 μm. (d) Appressorial formation rate of WT, Δ*SreC*, and Δ*SreC*‐C. (e) Symptoms of toxin produced by WT, Δ*SreC*, and Δ*SreC*‐C at 3 days post‐inoculation (dpi). (f) Toxin‐induced lesion size of WT, Δ*SreC*, and Δ*SreC*‐C. (g) The expression of melanin and cell wall‐degrading enzyme synthesis genes in WT, Δ*SreC*, and Δ*SreC‐*C at 48 hpi. The maize leaves were inoculated with WT, Δ*SreC* and Δ*SreC*‐C conidia at a concentration of 10^6^ conidia/mL. The leaves were sampled at indicated time for reverse transcription‐quantitative PCR assays. *C. lunata ClActin* was used as the reference gene. Values are means ± *SD* (*n* = 3 biological replicates). An asterisk indicates significant differences based on unpaired two‐tailed Student's *t* test with the *p* values marked (*p < 0.05, **p < 0.01, ns, not significant).

Our previous work revealed that the RIA pathway is involved in the formation of the infection structures in the biotrophic stage and that the SIA pathway regulates the activity of virulence factors in the necrotrophic stage during *C. lunata* infection (Lu et al., 2020). Notably, we found that all the conidia of WT CX‐3 and Δ*SreC*‐C strains germinated at 6 hpi, but the conidia of Δ*SreC* germinated at 10 hpi (Figure 6a,b), that is, the conidial germination of Δ*SreC* was obviously delayed. In addition, differentiation of appressoria in Δ*SreC* was significantly delayed compared to WT CX‐3 and Δ*SreC*‐C (Figure 6c), and there were significant differences in relative appressorial differentiation rates among WT CX‐3, Δ*SreC*‐C and Δ*SreC* (Figure 6d). These results indicated that SreC regulated the differentiation of infection structures in the biotrophic stage of *C. lunata*. Moreover, the lesion size caused by the nonhost‐selective toxin M5HF2C produced by Δ*SreC* was significantly smaller than that of WT CX‐3 and Δ*SreC*‐C (Figure 6e,f). We observed that the expression of biosynthesis genes for toxin M5HF2C and cell wall‐degrading enzymes genes were considerably down‐regulated in Δ*SreC* compared to WT CX‐3 and Δ*SreC*‐C (Figure 6g). These findings indicated that SreC regulated the cell wall‐degrading enzyme activity and M5HF2C biosynthesis in the necrotrophic stage of *C. lunata*. Taken together, the above results indicated that the decreased virulence of Δ*SreC* was due to the delayed lifestyle transition and impaired infection structures in the biotrophic stage and activity of virulence factors in the necrotrophic stage.

### 2.5 The function of Sre in adaption to host iron environments is conserved in hemibiotrophic fungi

**FIGURE 7:**
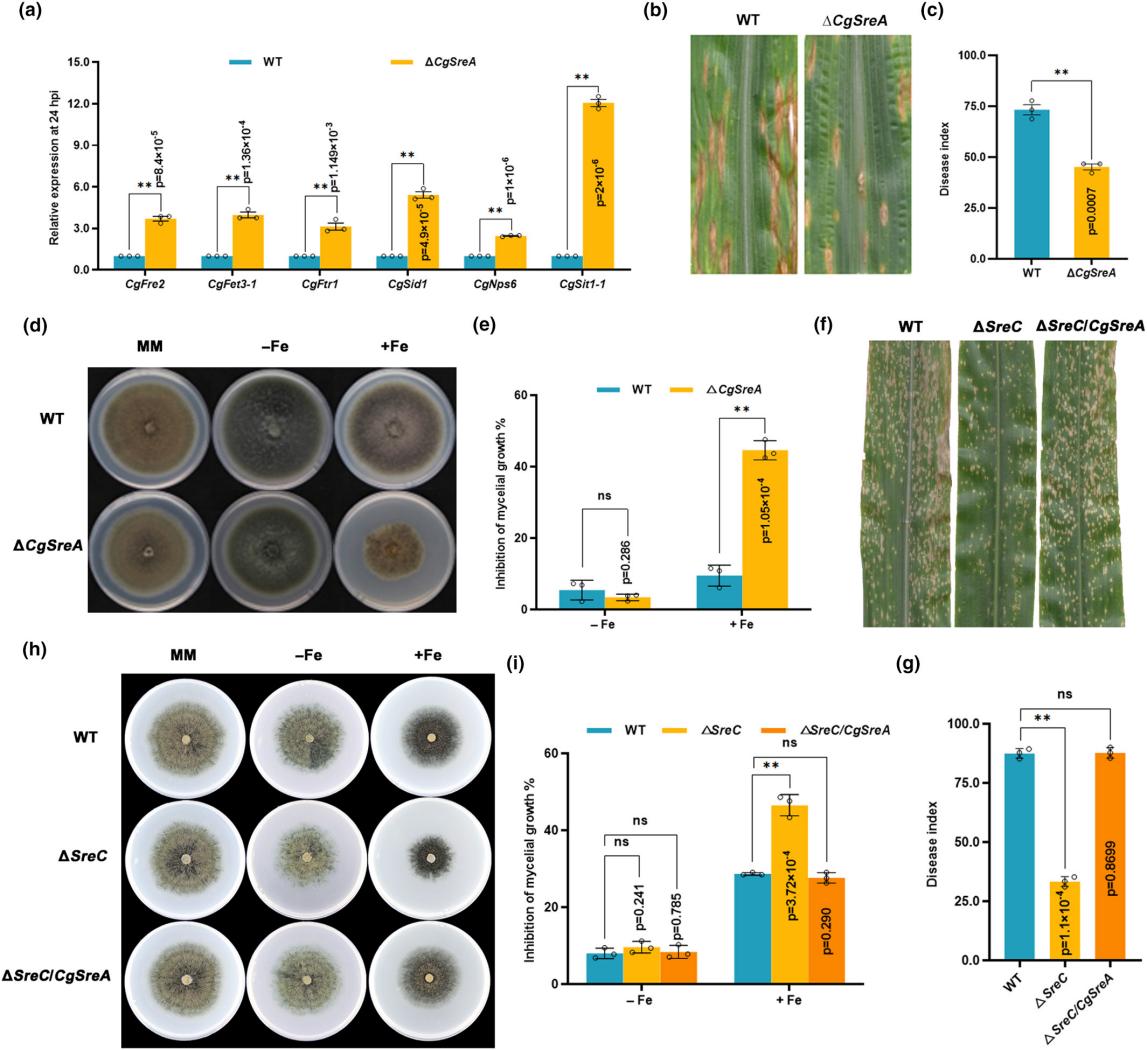
The function of Sre in adaption to host iron environments is conserved in hemibiotrophic fungi. (a) The expression of iron assimilation pathways genes in the wild type (WT) and Δ*CgSreA* at 24 h post‐inoculation (hpi). The maize leaves were inoculated with *Colletotrichum graminicola* conidia at a concentration of 10^4^ conidia/mL. The leaves were sampled at 24 hpi for reverse transcription‐quantitative PCR assays. *C. graminicola CgActin* was used as the reference gene. (b) Disease symptom of maize leaves infected with WT and Δ*CgSreA*. Conidial suspensions (10^4^ conidia/mL in 0.02% Tween 20) were sprayed onto the leave surfaces of eighth‐leaf stage maize seedlings. (c) Disease index of maize leaves infected with WT, Δ*CgSreA*. Others were as in (b). (d) Sensitivity of WT and Δ*CgSreA* to iron stress. A mycelial plug (5 mm) of each strain was inoculated on minimal medium (MM) with 50 μM bathophenanthroline disulfonate (BPS) (−Fe) or 1 mM FeCl_3_ (+Fe), then incubated at 28°C for 7 days. (e) Mycelial growth inhibition of WT and Δ*CgSreA* to iron stress. Mycelial growth inhibition of each treatment was calculated at 7 days post‐incubation (dpi). (f) Disease symptom of maize leaves infected with WT, Δ*SreC*, and Δ*SreC*/*CgSreA* at 7 dpi. Conidial suspensions (10^6^ conidia/mL in 0.02% Tween 20) were sprayed onto the leaf surfaces of maize at eighth‐leaf stage. (g) Disease index of maize leaves infected with WT, Δ*SreC*, and Δ*SreC*/*CgSreA*. Others were as in (f). (h) Sensitivity of WT, Δ*SreC*, and Δ*SreC*/*CgSreA* to iron stress. A mycelial plug (5 mm) of each strain was inoculated on MM with 50 μM BPS (−Fe) or 1 mM FeCl_3_ (+Fe), then incubated at 28°C for 7 days. (i) Mycelial growth inhibition of WT, Δ*SreC*, and Δ*SreC*/*CgSreA* to iron stress. Mycelial growth inhibition of each treatment was calculated 7 dpi. Values are means ± *SD* (*n* = 3 biological replicates). An asterisk indicates significant differences based on unpaired two‐tailed Student's *t* test with the *p* values marked (**p < 0.01, ns, not significant).

Phylogenetic analysis of Sre orthologues revealed high homology in filamentous fungi (Figure S6a), and thus the role of the Sre in *C. graminicola*, CgSreA, was also investigated in adaption to host iron environments. RT‐qPCR assays confirmed that the expression of genes in the RIA and SIA pathways were significantly up‐regulated in Δ*CgSreA* at 24 hpi (Figure 7a). Additionally, the virulence of the Δ*CgSreA* strain was significantly attenuated (Figure 7b,c). The Δ*CgSreA* displayed increased sensitivity to iron‐excess conditions (Figure 7d,e). These findings indicated that CgSreA was also essential for the adaption to the iron‐excess environment during *C. graminicola* infection. To further explore the functional conservation of Sre, we obtained the *CgSreA* complement in the Δ*SreC* strain (Figure S7a–c). In contrast to Δ*SreC*, defects in virulence and adaptation to an iron‐excess environment were restored in Δ*SreC/CgSreA* complemented strains (Figure 7f–i). These results revealed that the function of Sre in adaption to host iron environments is conserved.

**FIGURE 8:**
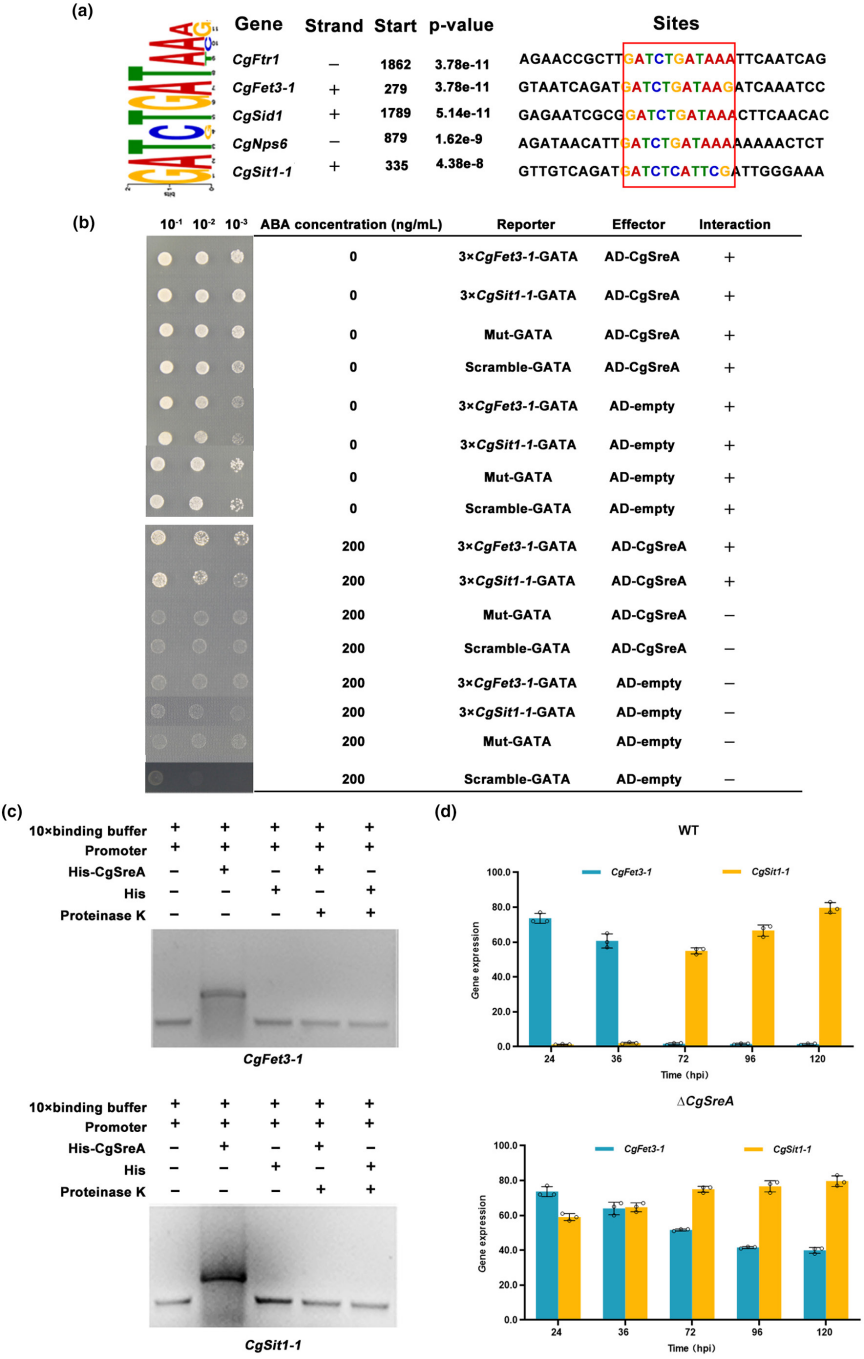
The function of Sre involved in the switch between iron assimilation pathways is conserved in hemibiotrophic fungi. (a) Identification of CgSreA binding motifs using the multiple EM for motif elicitation (MEME) program. The identified binding *cis*‐element in iron assimilation pathways genes is indicated in the red square. (b) Physical interaction of CgSreA with the promoters of *CgFet3‐1* and *CgSit1‐1* in yeast one‐hybrid system. Aureobasidin A (ABA) was added to the medium to inhibit the self‐activation of the *CgFet3‐1* and *CgSit1‐1* promoters. The negative control was mutant and scrambled GATA motif with AD‐CgSreA. The ABA (200 ng/mL) was added to synthetic dropout (SD) medium lacking leucine (L) (SD−L). (c) Verification of the binding of CgSreA with the promoters of *CgFet3‐1* and *CgSit1‐1* by electrophoretic mobility shift assay. The DNA probe was amplified using the *CgFet3‐1* and *CgSit1‐1* promoter regions containing the GATSTGATWMR element. His‐CgSreC was produced in *Escherichia coli* and purified. The DNA probe was incubated with purified His‐CgSreC and His with or without proteinase K for 20 min at 25°C. (d) Expression profiles of *CgFet3‐1* and *CgSit1‐1* during wild‐type (WT) and Δ*CgSreA* infection. The maize leaves were inoculated with *Colletotrichum graminicola* conidia at a concentration of 10^4^ conidia/mL. The leaves were sampled at indicated time for reverse transcription‐quantitative PCR assays. Values are means ± *SD* (*n* = 3 biological replicates).

The MEME analysis showed that genes in the RIA and SIA pathways of *C. graminicola* harboured the conserved CgSreA‐binding *cis*‐element GATSTGATWMR (where S = C/G, W = A/T, M = A/C, R = A/G) (Figure 8a). The results of Y1H and electrophoretic mobility shift (EMSA) assays showed that CgSreA could directly bind to the GATSTGATWMR motif of the promoters of *CgFet3‐1* and *CgSit1‐1*, of which the C‐terminal zinc finger was essential to perform this function (Figures 8b,c and S8a). In addition, Y1H and Y2H assays revealed that the iron‐binding of CgSreA was also vital for transcriptional regulation of the RIA and SIA pathways (Figure S8a,b). RT‐qPCR results showed that the absence of *CgSreA* also leads to an abnormal switch of the RIA to the SIA pathway (Figure 8d). Together with the findings from *C. lunata*, these findings suggested that Sre is involved in the switch from RIA to SIA pathways at the transcriptional level.

## 3 DISCUSSION

In plant–pathogen interactions, plants perturb the pathogenic iron homeostasis by localizing and targeting the redistribution of iron in infected plant tissues and cells (Expert et al., 1996; Verbon et al., 2017). Recruitment of iron in infection sites to wield against fungal pathogens is a critical immune response for *Poaceae* plants (Aznar et al., 2015; Greenshields et al., 2007). Consistently, our results showed that *C. lunata* infection provokes locally increased iron levels in host plant tissues after 24 hpi (Figure 1a), leading to drastic changes in the host iron environments. Moreover, we have demonstrated that SreC serves as a major regulator for adaption to host iron environments during *C. lunata* infection. Here, we observed that the expression of *SreC* was up‐regulated in response to local iron accumulation in host plants (Figure 1b). SreC subsequently bound to a ATGWGATAW motif in the promoter of genes in the RIA and SIA pathways and inhibited their expression in an iron‐dependent manner (Figures 2 and 3). Hemibiotrophic fungi switch RIA and SIA pathways to adapt to host iron environments at different trophic stages during infection (Albarouki & Deising, 2013; Liu et al., 2020). As demonstrated in our work, the switch between RIA and SIA pathways was abnormal in Δ*SreC* (Figure 4). SreC has multiple roles in adaption to the host iron environment during *C. lunata* infection, for example, the absence of *SreC* also led to impaired transition of trophic stages and developmental processes (Figures 5 and 6). CgSreA has similar functions in adaption to the host iron environment and virulence in *C. graminicola* (Figures 7 and 8), suggesting the role of Sre is conserved in hemibiotrophic fungi. The line of evidence presented in this study indicates that Sre‐dependent adaption to host iron environments is essential to the infectious growth and survival of hemibiotrophic fungi within the host plant. Therefore, we present a regulatory mechanism of host plant iron environment responses mediated by SreC during fungal infection, providing insights into how hemibiotrophic fungi adapt to host environments during infection.

Maintaining the balance of intracellular iron levels is indispensable to ensure optimal cellular metabolism and virulence in pathogens. Sre is a core transcriptional regulator that represses both RIA and SIA pathways during iron sufficiency to avoid iron overload in pathogenic fungi (Zhang et al., 2013). In mammal‐pathogenic fungi, Sre orthologues have no role in virulence (Chen et al., 2011; Schrettl et al., 2008). However, its role in virulence is substantially changed in plant‐pathogenic fungi, such as *U. maydis*, *C. heterostrophus*, and *F. graminearum* (An, Mei, et al., 1997; An, Zhao, et al., 1997; Wang et al., 2019; Zhang et al., 2013). However, in *A. alternata*, the Sre orthologue is also involved in the response to oxidative stress resistance and cell wall integrity but has a dispensable role in virulence (Chung et al., 2020). In our study, Sre regulated adaption to host iron environments during infection in the hemibiotrophic fungi *C. lunata* and *C. graminicola*. We conclude that Sre has conserved functions in the regulation of iron homeostasis, but the regulation of virulence is variable.

A unique conjugation between the iron high‐affinity uptake pathways and lifestyles exists in pathogenic fungi, blocking iron uptake pathways showed varying virulence profiles in maize for different trophic types of phytopathogenic fungi. The biotrophic and necrotrophic fungi have been found to rely on RIA and SIA pathways for iron acquisition, respectively, in the infection process (Condon et al., 2014; Eichhorn et al., 2006; Mei et al., 1993; Oide et al., 2006). Hemibiotrophic fungi used RIA and SIA pathways in biotrophic and necrotrophic stages, respectively (Albarouki et al., 2014). In our study, RIA and SIA pathways also specifically correspond to the biotrophic and necrotrophic stages during *C. lunata* infection (Figure 4a). These results demonstrate that upon sensing the host iron environments, hemibiotrophic fungi undergo dramatic transcriptional remodelling to switch their iron assimilation pathways to ensure full virulence during their infectious cycle, but the activation mechanism is unknown. In fungi, the activation of RIA and SIA pathways is via suppressing Sre during infection (Chung et al., 2020; Pelletier et al., 2007; Schrettl et al., 2008; Zhang et al., 2013). In this study, the absence of *SreC* only resulted in anomalous switching of RIA and SIA pathways rather than non‐transition (Figure 4). These results demonstrate that the switch of iron assimilation pathways during hemibiotrophic fungi infection is the result of the regulation of multiple transcription factors or involves the regulation of posttranslational and chromatin levels.

It remains unclear how these fungal trophic stages transition and how plant signals elicit the transition of trophic stages. In *Colletotrichum lindemuthianum*, CLNR1 functions as the AREA/NIT2‐like global nitrogen regulator, the *clnr1* mutant is impaired during the initiation of the necrotrophic stage (O'Connell et al., 2012; Pellier et al., 2003). In *Magnaporthe oryzae*, PacC signalling pathway is necessary for regulating the switch in the fungal lifestyle associated with rice blast (Chen et al., 2020). These results show that nitrogen starvation and cellular pH changes can act as signals for the regulation of lifestyle transition. The change of iron status in host cells affects the course of disease pathogenesis during hemibiotrophic fungal infection. For instance, *C. graminearum* quickly switches from the biotrophic stage to the necrotrophic stage and induces host plant cell death when maize leaves are iron deficient (Albarouki et al., 2014; Albarouki & Deising, 2013). The infectious structure differentiation of *C. graminearum* in the biotrophic stage is inhibited under sufficient‐iron conditions in maize leaves (Ye et al., 2014). In this study, we found a significant difference in the iron content of the infected host cells in the biotrophic and necrotrophic stages of *C. lunata* (Figure 1a), indicating that host cellular iron may act as a signal for the trophic stage switch in hemibiotrophic fungi. Here, we found that the switch from the biotrophic stage to the necrotrophic stage in the *SreC* mutant was significantly delayed (Figure 5a,b), which may be due to the absence of SreC leading to the inability of sense the host iron environment.

In summary, our study on fungal transcription factors SreC provides molecular insights into understanding the mechanisms of adaptation to the host plant iron environment, which is crucial for the transition of trophic stages and developmental processes in *C. lunata,* and the function is conserved in hemibiotrophic fungi. This study reveals a novel regulatory mechanism of fungal pathogens to adapt to dynamic host environments during infection.

## 4 EXPERIMENTAL PROCEDURES

### 4.1 Fungal strains, plant materials, and culture conditions

*C. lunata* strain CX‐3 and *C. graminicola* strain M1.001 were used as the wild type (WT) strains. The Δ*SreC*, Δ*SreC‐*C, Δ*SreC/CgSreA* in *C. lunata* and Δ*CgSreA* in *C. graminicola* were generated using the homologous recombination approach by Zhang et al. (2016). All the conidial suspensions were stored at −80°C in 25% glycerol. All the strains were initially grown on potato dextrose agar (PDA) plates (Solarbio). Mycelial growth was assayed on minimal medium (MM, 10 g glucose, 10 mL solution A contains 1 g Ca(NO_3_)_2_·4H_2_O, 10 mL solution B contains 0.2 g KH_2_PO_4_, 0.25 g MgSO_4_·7H_2_O, 0.15 g NaCl, 500 μL solution SRB contains 28.6 μg H_3_BO_3_, 196.5 μg CuSO_4_·5H_2_O, 6.55 μg KI, 30.2 μg MnSO_4_·H_2_O, 18.4 μg (NH_4_)_6_MO_7_O_24_·4H_2_O, 2745 μg ZnSO_4_·H_2_O, 474.1 FeCl_3_·6H_2_O and 20 g agar L^−1^), and the colony diameter was measured at 7 days post‐incubation (dpi). Conidiation and conidia germination in MM were assayed. The maize inbred line B73 grew to the eighth‐leaf stage, and the barley cultivar Zaoshusan was grown to the fifth‐leaf stage in a growth chamber at 24°C under 14 h of light/10 h of dark before conducting the inoculation assay.

### 4.2 Prussian blue staining for Fe^3+^ detection

Histochemical staining of Fe^3+^ was performed as described previously with slight modifications (Liu et al., 2007). To visualize the iron status in barley epidermal cells at infection sites, the Prussian blue assay used barley leaf tissues treated with acid solutions of ferrocyanides. Thin epidermal layers of barley leaves inoculated with *C. lunata* strains were incubated in 7% potassium ferrocyanide and 2% hydrochloric acid (HCl) (1:1, vol/vol) for 15 h at 25°C. Fe^3+^ inside the leaf epidermal cells combines with the ferrocyanides, which results in the formation of bright blue pigments.

### 4.3 Gene expression analysis

Total RNA was extracted using the EasyPure plant RNA Kit (TransGen Biotech), and cDNA was synthesized from the RNAs using the HiScript II 1st Strand cDNA Synthesis Kit (Vazyme). RT‐qPCR was performed with the AceQ Universal SYBR qPCR Master Mix (Vazyme) and an ABI PRISM 7900HT system (Applied Biosystems). Each gene was assayed independently and in triplicate. Relative expression ratios were calculated using the ΔΔ*C*_t_ method (Livak & Schmittgen, 2001). All primers used in this assay are listed with brief descriptions in Table S1.

### 4.4 Virulence assays

Maize plants were inoculated with 2 mL conidial suspension (10^6^ conidia/mL) per plant and placed in a humidity chamber (relative humidity at 100%) in the greenhouse for 48 h following inoculation. The greenhouse was maintained at 28°C in daytime and 18°C at night. All the inoculated plants were observed after 7 days and checked for disease levels. Disease severity was evaluated as described by Zhang et al. (2019). Photographed leaves were imaged using Photoshop CS5.

### 4.5 Bioinformatics for binding motif prediction

Promoter sequences of the genes were obtained from the NCBI database, and then the transcription start site was predicted using Promoter v. 2.0. Promoter sequences of the genes were analysed by searching binding‐site motifs using MEME v. 5.5.5. Briefly, 2000 bp DNA sequences before the transcriptional start site were predicted and analysed using the 0 or 1 occurrence per sequence constraint.

### 4.6 Y1H assay

To construct plasmids for Y1H analysis, the DNA fragments containing three repeats of GATA‐boxes (5′‐ATCWGATAW‐3′) in RIA/SIA pathway genes promoter were synthesized and inserted into the reporter vector pAbAi (Clontech) to generate the pAbAi‐bait plasmids. To test the specificity of the binding sites, fragments carrying the same flanking regions with GATA‐box elements mutated (i.e., replaced with 5′‐ATCWGCAAW‐3′) were synthesized and inserted into the pAbAi reporter vector to generate the pAbAi‐mutated plasmid. The full length *SreC* and *CgSreA* coding sequences were amplified by PCR and inserted into the pGADT7 vector. The recombinant pGADT7‐SreC and pGADT7‐CgSreA plasmids were used to transform Y1HGold yeast strain cells carrying the linearized pAbAi‐bait and pAbAi‐mutant. Transformed yeast cells were detected by spotting serial dilutions of yeast onto agar‐solidified synthetic dropout (SD)/−Leu medium supplemented with aureobasidin A (ABA, 20 ng/mL). Details of the primers used for this assay are listed in Table S1.

### 4.7 Dual‐luciferase reporter assay

The promoter fragments of *ClFet3p* and *ClSit1p* in RIA and SIA pathways were cloned into the pGreenII 0800‐LUC vector, and the full‐length coding region of the *SreC* gene was cloned into the pGreenII 62‐SK vector. The recombinant plasmids were transformed into *Agrobacterium tumefaciens* GV3101 cells, and the cultured *Agrobacterium* was injected into *N. benthamiana* (Yao et al., 2009). Injected leave tissue was harvested 3 days after injection and frozen in liquid nitrogen. Extracted RNA was used to analyse the relative LUC/REN expression. Primer sequences are given in Table S1.

### 4.8 Electrophoretic mobility shift assay

The cDNA encoding the proteins SreC and CgSreA were amplified and cloned into the pET‐28a(+) vector to generate a His‐tagged protein. The resulting construction vector was transformed into *E. coli* BL21(DE3) after the cDNA was sequenced. The cells were grown in Luria Bertani (LB) medium at 37°C to OD_600_ of 0.6. Expression of genes was then induced by the addition of 1 mM isopropyl β‐d‐1‐thiogalactopyranoside (IPTG), with 1 mM FeCl_3_ and 50 μm BPS, and the continued incubation at 37°C for additional 3 h. The recombinant proteins were purified by Ni‐sepharose beads and eluted by reduced glutathione. Promoter sequences are amplified using the primers specified in Table S1. For EMSA, reaction mixtures containing purified His‐SreC or His‐CgSreA, promoter DNAs, and 10× binding buffer (100 mM Tris–HCl pH 7.5, 0.5 M NaCl, 10 mM dithiothreitol, 10 mM EDTA, 50% glycerol) were incubated for 20 min at 25°C (Wang et al., 2019). Purified His was used as a negative control. The reactions were electrophoresed on 1.2% agarose gel in 0.5 × Tris‐acetate‐EDTA buffer for 45 min at 80 V under low temperature. Signals were detected in the J3‐3000 imaging system after staining the DNA with ethidium bromide for 15 min.

### 4.9 Infection process and lifecycle transition observations

Leaves of barley were droplet inoculated with 3 μL of conidial suspension (10^4^ conidia/mL) onto the adaxial surface without damaging and were allowed to develop necrotic lesions. Infection processes were observed under the light microscope at 6, 12, 24, 36, 48, and 72 hpi at 25°C. To visualize the viability of barley epidermal cells at infection sites and determine the trophic stage of the fungal pathogen, trypan blue staining of barley leaves was performed, as described previously by Lipka et al. (2005). All microscopic observations were made with a laser‐scanning confocal microscope (FV3000; Olympus).

### 4.10 Y2H assay

To construct plasmids for Y2H analysis, the coding sequence of each gene was amplified from cDNA of the *C. lunata* CX‐3 with the corresponding primer pairs listed in Table S1. Each cDNA fragment was cloned into the yeast GAL4‐binding domain vector pGBKT7 and GAL4‐activation domain vector pGADT7 (Clontech). Pairs of Y2H plasmids were co‐transformed into yeast strain AH109 following the lithium acetate/single‐stranded DNA/polyethylene glycol transformation protocol. The plasmid pairs pGBKT7‐53 and pGADT7‐T were used as positive controls; the plasmid pairs pGBKT7‐Lam and pGADT7‐T were used as negative controls. Transformants were grown at 30°C for 3 days on synthetic dropout (SD) medium lacking Leu and Trp and then transferred to SD without His, Leu, Trp, and Ade to assess protein–protein interaction.

### 4.11 BiFC assay

BiFC assays were performed as described previously (Kamigaki et al., 2016). The coding sequences of SreC, ClGrx4, and ClFra2 were constructed in‐frame with YN (N‐terminal YFP) and/or YC (C‐terminal YFP). Each plasmid was introduced into *Agrobacterium tumefaciens* GV3101, and then appropriate combinations of BiFC constructs with the nuclear localization signal‐targeted H2B‐RFP plasmid were co‐infiltrated into the abaxial side of 4‐week‐old *N. benthamiana* leaves with a 1 mL needleless syringe. Fluorescence was visualized with a confocal laser‐scanning microscope (FV3000; Olympus) using the preset settings for YFP (excitation: 514 nm, emmission: 510–550 nm) and H2B‐RFP (excitation: 557 nm, emmission: 579–586 nm) after 48 h.

### 4.12 Toxin activity assays

Flasks containing 100 mL Fries medium (2 g KNO_3_, 0.5 g KCl, 1 mg FeSO_4_, 1 g K_2_HPO_4_, 0.5 g MgSO_4_·7H_2_O, 0.2 mg VB1, 0.5 g l‐aspartic acid L^−1^) were inoculated with mycelial plugs and incubated on a rotary shaker at 25°C for 15 days. The filtrate was concentrated to approximately 10% of the original volume at 40°C using a vacuum rotary evaporator (Senco). An equal volume of methanol was supplemented, and the samples were stored overnight at 4°C. The filtrate was evaporated under vacuum at 40°C to remove the methanol, and the aqueous fraction was extracted three times. The residues were weighed and adjusted to 1 mg/mL with deionized water. The activity of the toxin was tested on the fourth leave of maize seedlings at the eight‐leaf stage. Puncture wounds were made on the upper surface of the leaves, and 10 μL of the toxin was dropped onto the wounded and intact leaves of maize inbred line B73 by pipette. The treated leaves were placed in Petri dishes lined with moistened filter papers with 10 mM 6‐benzylaminopurine, then incubated at 28°C for 3 days (Alam et al., 1997).

## CONFLICT OF INTEREST STATEMENT

The authors declare that the research was conducted without any commercial or financial relationships that could be construed as a potential conflict of interest.

## Supporting information


**FIGURE S1.** Domain of the SreC revealed high similarity to its orthologues. (a) Phylogenetic tree of SreC and its orthologues. All the amino acid sequences were aligned using ClustalW and the phylogenetic tree was constructed using MEGA 6 BETA. All the Sre protein sequences were downloaded from the NCBI database. (b) Amino acid sequences alignment of two zinc finger (ZnF1, 2) and cysteine‐rich central (CRR) domains of SreC orthologues.

**FIGURE S2.** Strategy of *SreC* deletion and complementation, and verification of mutant and complementation strains. (a) Gene replacement strategy for Δ*SreC* and Δ*SreC‐*C. (b) Colony morphology of *SreC* gene knockout and complementary mutants. The wild type (WT) and mutant strains were grown in minimal medium for 7 days. (c) *SreC* gene knockout mutant verification by PCR analysis. M, Trans 5 K Marker. CX‐3, 1, 4, 7, 10. Δ*SreC*, 2, 3, 5, 6, 8, 9, 11, 12. (d) *SreC* gene complementary mutant verification by PCR analysis. M, Trans 5K Marker. CX‐3, 1, 4, 7, 10. Δ*SreC*‐C, 2, 3, 5, 6, 8, 9, 11, 12.

**FIGURE S3.** SreC binds to the promoters of reductive iron assimilation (RIA) and siderophore‐mediated iron assimilation (SIA) pathways genes. (a) High‐affinity iron assimilation pathways for *Curvularia lunata*. RIA pathway occurred on the plasma membrane of *C. lunata*, extracellular Fe^3+^ was reduced to Fe^2+^ by ferric reductase ClFreB, Fe^2+^ by multicopper oxidase ClFet3p was oxidized to Fe^3+^, which was transported into the cell by iron permease ClFtr1. SIA pathway used L‐ornithine‐N‐5‐oxygenase ClSidA and non‐ribosomal peptide synthetase ClNps6 synthesizes siderophores and secreted them into extracellular to chelate iron to form siderophores‐ Fe^3+^, which was transported into the cell via a specific transporter ClSit1p and releases Fe^3+^. (b) Physical interaction of SreC with the promoters of RIA and SIA pathways genes in the yeast one‐hybrid hybridization system. Aureobasidin A (ABA) was supplemented into the medium to inhibit the self‐activation of the RIA and SIA pathway gene promoters. The negative control was genes promoter‐bait with AD. The concentration of ABA supplemented in synthetic dropout (SD) medium lacking leucine (L) (SD−L) was 200 ng/mL.

**FIGURE S4.** ClGrx4 and ClFra2 are required for the function of adaption to the host iron excess environment of *Curvularia lunata* during infection. (a) ClGrx4 interacted with ClFra2 in yeast two‐hybrid assay. Serial dilutions of the yeast cells were plated on synthetic dropout (SD) medium lacking leucine (L), tryptophan (T), histidine (H), and adenine (A) (SD−L−T−H−A). The yeast strain containing pGBKT7‐53 and pGADT7 was used as a positive control, containing pGBKT7‐Lam and pGADT7 was used as a negative control. (b) Δ*ClCrx4* and Δ*ClFra2* exhibited increased sensitivity to iron excess. A mycelial plug (5 mm) of each strain was inoculated on minimal medium with 50 μM bathophenanthroline disulfonate (BPS) (−Fe) and 1 mM FeCl_3_ (+Fe) and then incubated at 28°C for 7 days. (c) Mycelial growth inhibition of wild type (WT), Δ*ClGrx4*, and Δ*ClFra2* to iron stress. Mycelial growth inhibition of each treatment was calculated after 7 days post‐incubation. (d) Expression profiles of *ClFet3p* and *ClSit1p* during Δ*ClGrx4* and Δ*ClFra2* infection. The maize leaves were inoculated with Δ*ClGrx4* and Δ*ClFra2* conidia at a concentration of 10^6^ conidia/mL. The leaves were sampled at the indicated time for reverse transcription‐quantitative PCR assays. The *C. lunata ClActin* was used as a reference gene. hpi, hours post‐inoculation. Values are means ± *SD* (*n* = 3 biological replicates). An asterisk indicates significant differences based on unpaired two‐tailed Student’s *t* test with the *p* values marked (*p < 0.05, **p < 0.01, ns, not significant).

**FIGURE S5** Construction of *ClFet3p*‐eGFP and *ClSit1p*‐eGFP fusion expression strains. (a) The *ClFet3p*‐eGFP and *ClSit1p*‐eGFP fusion expression strains displayed no defect in colony morphology. The wild type (WT), *ClFet3p*‐eGFP, and *ClSit1p*‐eGFP strains were grown on minimal medium (MM) for 7 days and photographed. (b) PCR analysis of the WT, *ClFet3p*‐eGFP and *ClSit1p*‐eGFP strains. M, Trans 5K Marker; *ClFet3p*‐eGFP strain, 1, 2. *ClSit1p*‐eGFP strain, 3, 4, 5. (c) Control of *ClFet3p* and *ClSit1p* expression by the availability of iron, as measured by the eGFP‐fluorescence of *ClFet3p*‐eGFP and *ClSit1p*‐eGFP fusion expression strains. Conidia of each strain were cultured on MM with 50 μM bathophenanthroline disulfonate (BPS) (−Fe) and 1 mM FeCl_3_ (+Fe), then incubated at 28°C for 7 days. (d) The fluorescence intensity of *ClFet3p*‐eGFP and *ClSit1p*‐eGFP strains at low and high iron stress. Values are means ± *SD* (*n* = 3 biological replicates). An asterisk indicates significant differences based on unpaired two‐tailed Student’s *t* test with the *p* values marked (*p < 0.05, **p < 0.01, ns, not significant).

**FIGURE S6** Strategy of *CgSreA* deletion and verification of mutant strains. (a) Phylogenetic tree of CgSreA and its orthologues. All the amino acid sequences were aligned using the ClustalW program and the phylogenetic tree was constructed using MEGA 6 BETA program. All Sre protein sequences were downloaded from the NCBI database. (b) Gene replacement strategy for the deletion *CgSreA*. (c) Colony of wild type (WT) M1.001 and Δ*CgSreA* grown on minimal medium at 25°C for 7 days. (d) *CgSreA* gene knockout mutants Verification by PCR analysis. M, Trans 5K Marker. CX‐3, 1, 4, 7, 10. Δ*CgSreA*, 2, 3, 5, 6, 8, 9.

**FIGURE S7** Strategy of the *CgSreA* complement in the Δ*SreC* strain and verification Δ*SreC/CgSreA* strain. (a) Gene replacement strategy for Δ*SreC/CgSreA*. (b) Colony morphology of *SreC* gene knockout and Δ*SreC/CgSreA* complementary mutants. The wild‐type (WT) and mutant strains were grown in minimal medium for 7 days. (c) Δ*SreC/CgSreA* strain verification by PCR analysis. M, Trans 5K Marker. CX‐3, 4, 8, 12. Δ*SreC/CgSreA*, 1, 2, 3, 5, 6, 7, 9, 10, 11.

**FIGURE S8** CgSreA has similar function in *Colletotrichum graminicola*. (a) The C‐terminal zinc finger and cysteine‐rich central domains of CgSreA are required to interact with the promoters of genes in reductive iron assimilation (RIA) and siderophore‐mediated iron assimilation (SIA) pathways in yeast one‐hybrid hybridization system. Aureobasidin A (ABA) was added to the medium to inhibit the self‐activation of the *CgSit1‐1* promoters. The positive control was *CgSit‐1* promoter‐bait with AD‐CgSreA. The negative control was *CgSit1‐1* promoter‐bait with AD. Concentration of ABA supplemented into synthetic dropout (SD) medium lacking leucine (L) (SD−L) was 200 ng/mL. (b) CgSreA interacted with CgGrx4 and CgFra2 in yeast two‐hybrid hybridization assay. Serial dilutions of the yeast cells were plated on SD medium lacking leucine (L), tryptophan (T), histidine (H), and adenine (A) (SD−L−T−H−A). The yeast strain containing pGBKT7‐53 and pGADT7 was used as a positive control, whereas that containing pGBKT7‐Lam and pGADT7 was used as a negative control.

**TABLE S1.** Primers used in this study.

## Data Availability

The authors declare that all data supporting the findings of this study are available within the paper and the supplementary files or are available from the corresponding author upon request.
