# Supplementary figures and images for: SreC‐dependent adaption to host iron environments regulates the transition of trophic stages and developmental processes of Curvularia lunata
Source: Mol Plant Pathol. 2024 Mar 13;25(3):e13444. doi: 10.1111/mpp.13444 (PMC10938068; doi:10.1111/mpp.13444)

(a)

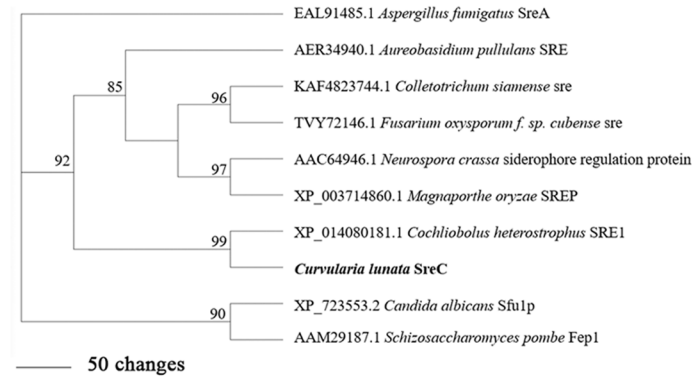

(b)

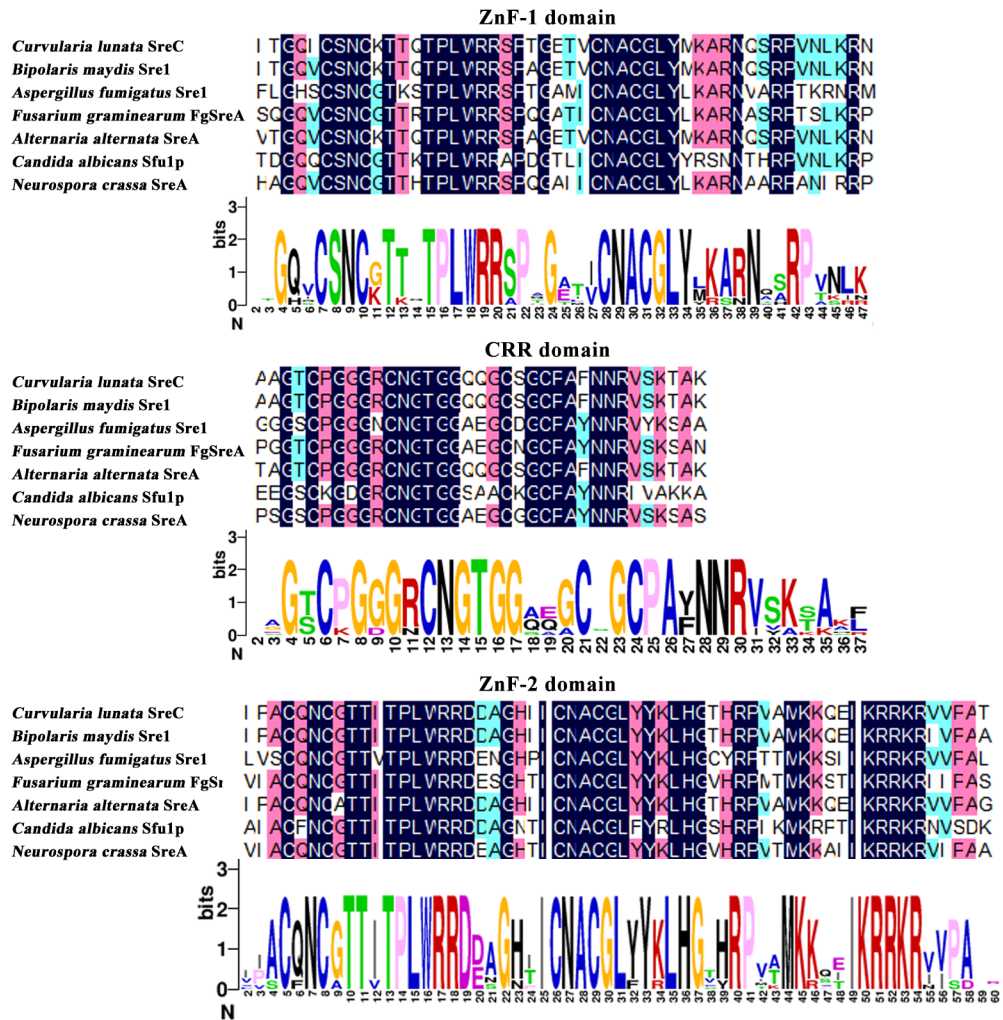

Supplement: Supplementary file 1 — FIGURE S1. Domain of the SreC revealed high similarity to its orthologues. (a) Phylogenetic tree of SreC and its orthologues. All the amino acid sequences were aligned using ClustalW and the phylogenetic tree was constructed using MEGA 6 BETA. All the Sre protein sequences were downloaded from the NCBI database. (b) Amino acid sequences alignment of two zinc finger (ZnF1, 2) and cysteine‐rich central (CRR) domains of SreC orthologues. [file MPP-25-e13444-s001.pdf]

(a)

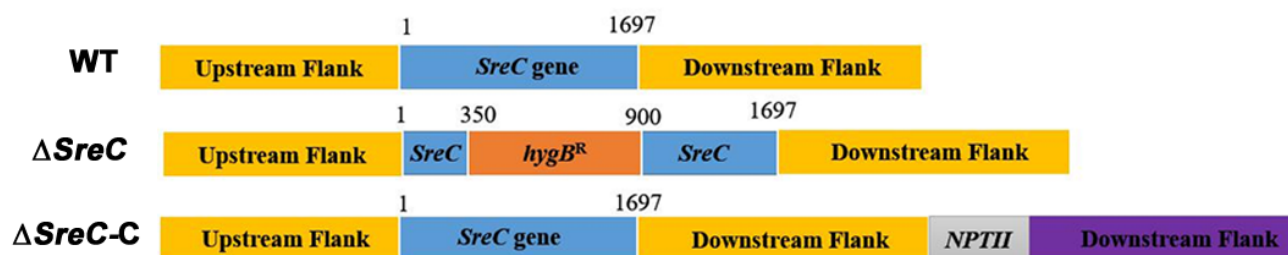

(b)

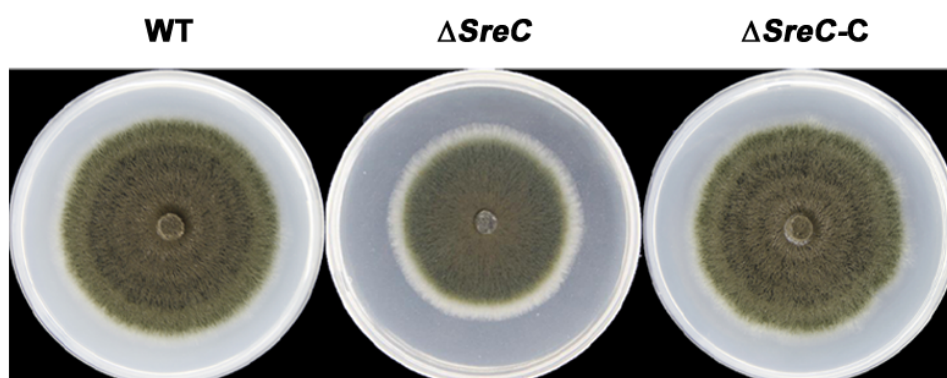

(c)

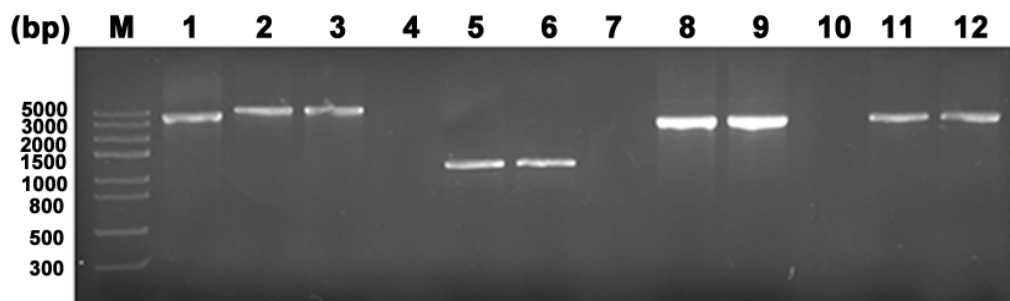

(d)

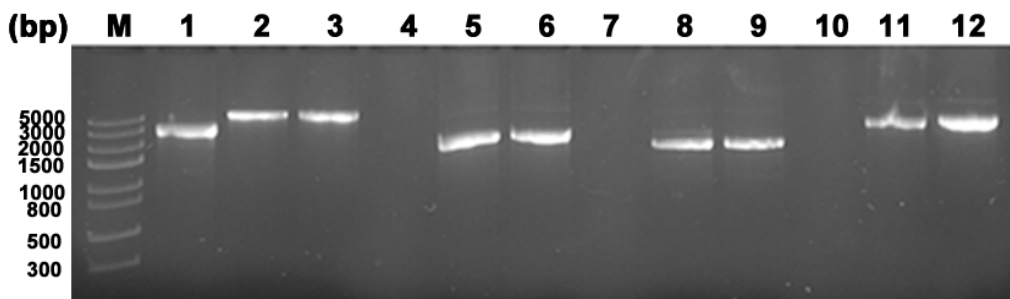

Supplement: Supplementary file 2 — FIGURE S2. Strategy of SreC deletion and complementation, and verification of mutant and complementation strains. (a) Gene replacement strategy for ΔSreC and ΔSreC‐C. (b) Colony morphology of SreC gene knockout and complementary mutants. The wild type (WT) and mutant strains were grown in minimal medium for 7 days. (c) SreC gene knockout mutant verification by PCR analysis. M, Trans 5 K Marker. CX‐3, 1, 4, 7, 10. ΔSreC, 2, 3, 5, 6, 8, 9, 11, 12. (d) SreC gene complementary mutant verification by PCR analysis. M, Trans 5K Marker. CX‐3, 1, 4, 7, 10. ΔSreC‐C, 2, 3, 5, 6, 8, 9, 11, 12. [file MPP-25-e13444-s002.pdf]

(a)

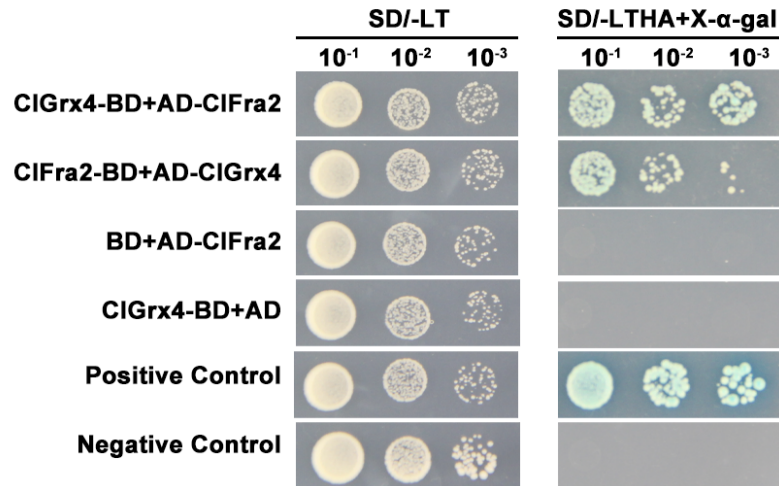

(b)

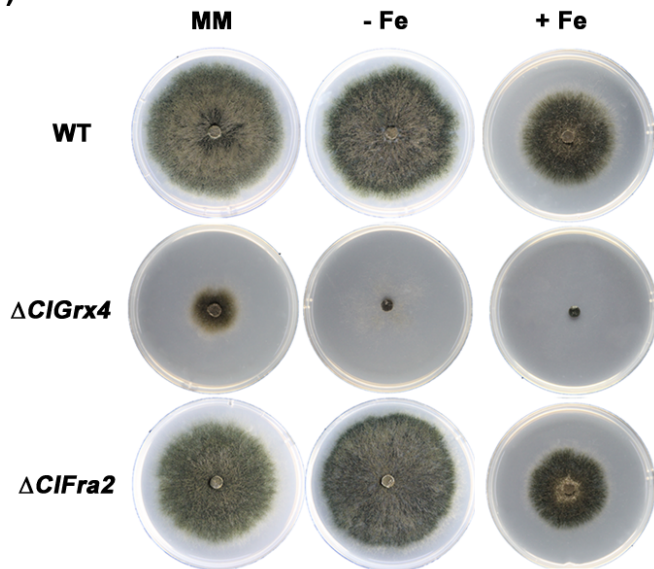

(c)

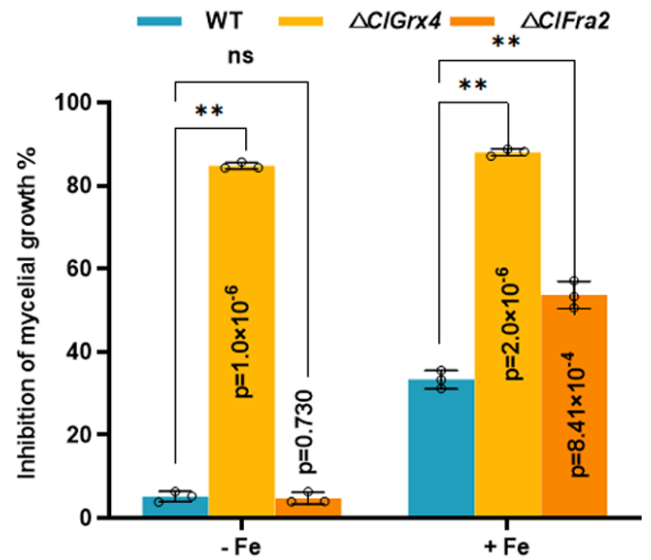

(d)

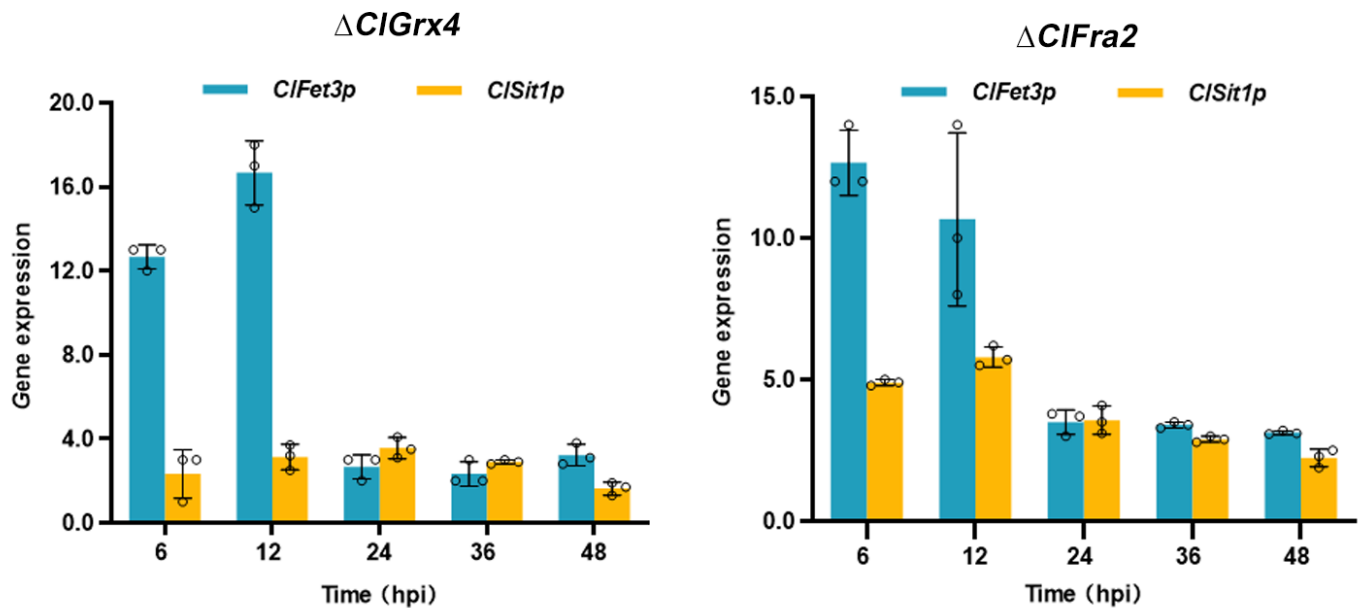

Supplement: Supplementary file 4 — FIGURE S4. ClGrx4 and ClFra2 are required for the function of adaption to the host iron excess environment of Curvularia lunata during infection. (a) ClGrx4 interacted with ClFra2 in yeast two‐hybrid assay. Serial dilutions of the yeast cells were plated on synthetic dropout (SD) medium lacking leucine (L), tryptophan (T), histidine (H), and adenine (A) (SD−L−T−H−A). The yeast strain containing pGBKT7‐53 and pGADT7 was used as a positive control, containing pGBKT7‐Lam and pGADT7 was used as a negative control. (b) ΔClCrx4 and ΔClFra2 exhibited increased sensitivity to iron excess. A mycelial plug (5 mm) of each strain was inoculated on minimal medium with 50 μM bathophenanthroline disulfonate (BPS) (−Fe) and 1 mM FeCl3 (+Fe) and then incubated at 28°C for 7 days. (c) Mycelial growth inhibition of wild type (WT), ΔClGrx4, and ΔClFra2 to iron stress. Mycelial growth inhibition of each treatment was calculated after 7 days post‐incubation. (d) Expression profiles of ClFet3p and ClSit1p during ΔClGrx4 and ΔClFra2 infection. The maize leaves were inoculated with ΔClGrx4 and ΔClFra2 conidia at a concentration of 106 conidia/mL. The leaves were sampled at the indicated time for reverse transcription‐quantitative PCR assays. The C. lunata ClActin was used as a reference gene. hpi, hours post‐inoculation. Values are means ± SD (n = 3 biological replicates). An asterisk indicates significant differences based on unpaired two‐tailed Student’s t test with the p values marked (*p < 0.05, **p < 0.01, ns, not significant). [file MPP-25-e13444-s003.pdf]

(a)

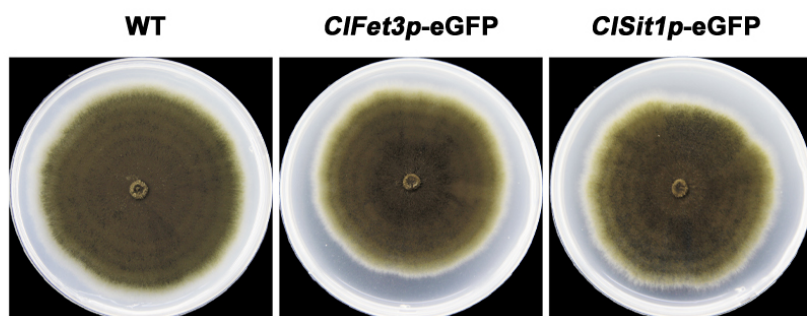

(b)

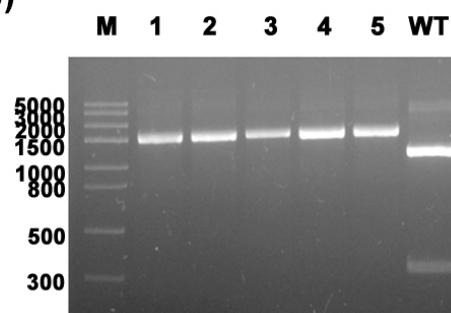

(c)

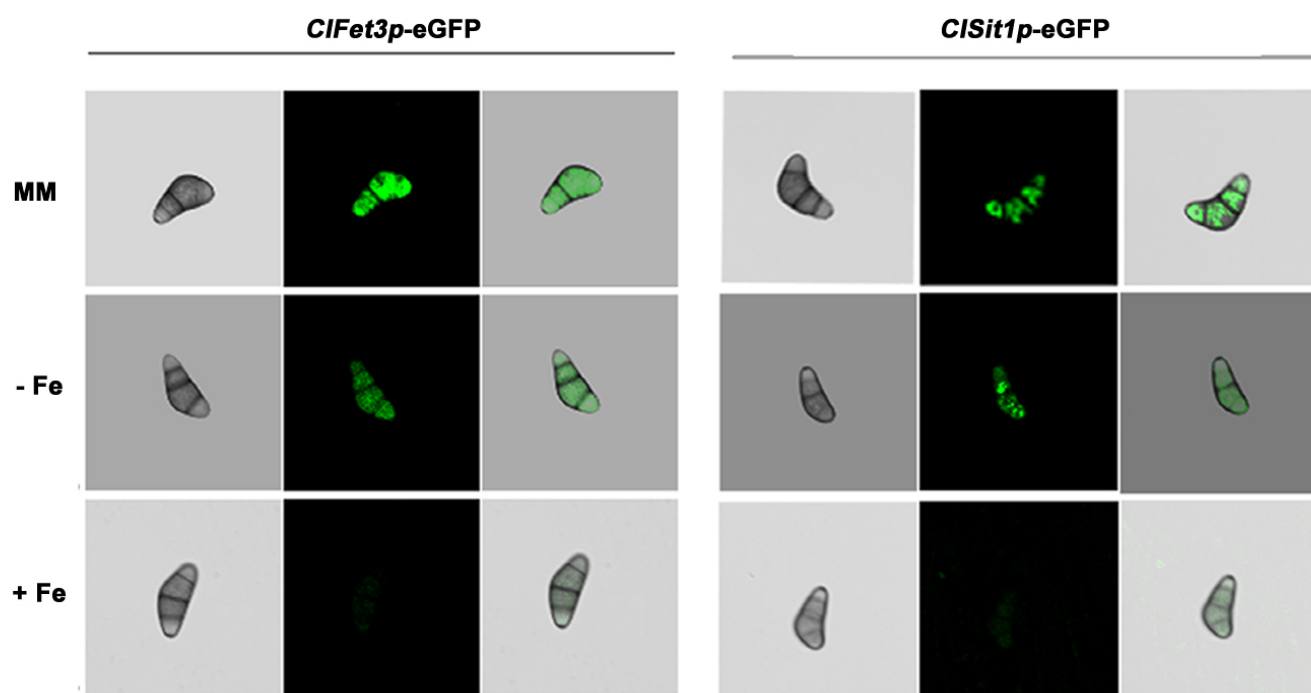

(d)

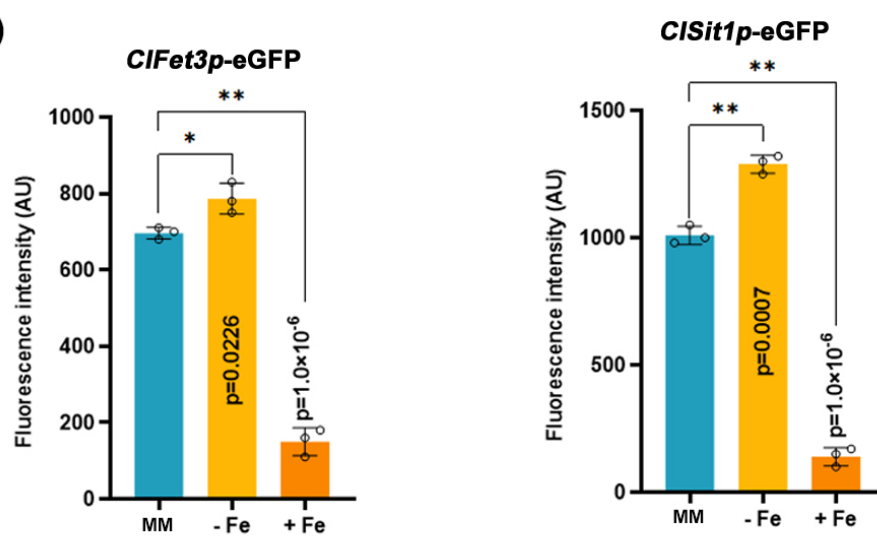

Supplement: Supplementary file 5 — FIGURE S5 Construction of ClFet3p‐eGFP and ClSit1p‐eGFP fusion expression strains. (a) The ClFet3p‐eGFP and ClSit1p‐eGFP fusion expression strains displayed no defect in colony morphology. The wild type (WT), ClFet3p‐eGFP, and ClSit1p‐eGFP strains were grown on minimal medium (MM) for 7 days and photographed. (b) PCR analysis of the WT, ClFet3p‐eGFP and ClSit1p‐eGFP strains. M, Trans 5K Marker; ClFet3p‐eGFP strain, 1, 2. ClSit1p‐eGFP strain, 3, 4, 5. (c) Control of ClFet3p and ClSit1p expression by the availability of iron, as measured by the eGFP‐fluorescence of ClFet3p‐eGFP and ClSit1p‐eGFP fusion expression strains. Conidia of each strain were cultured on MM with 50 μM bathophenanthroline disulfonate (BPS) (−Fe) and 1 mM FeCl3 (+Fe), then incubated at 28°C for 7 days. (d) The fluorescence intensity of ClFet3p‐eGFP and ClSit1p‐eGFP strains at low and high iron stress. Values are means ± SD (n = 3 biological replicates). An asterisk indicates significant differences based on unpaired two‐tailed Student’s t test with the p values marked (*p < 0.05, **p < 0.01, ns, not significant). [file MPP-25-e13444-s008.pdf]

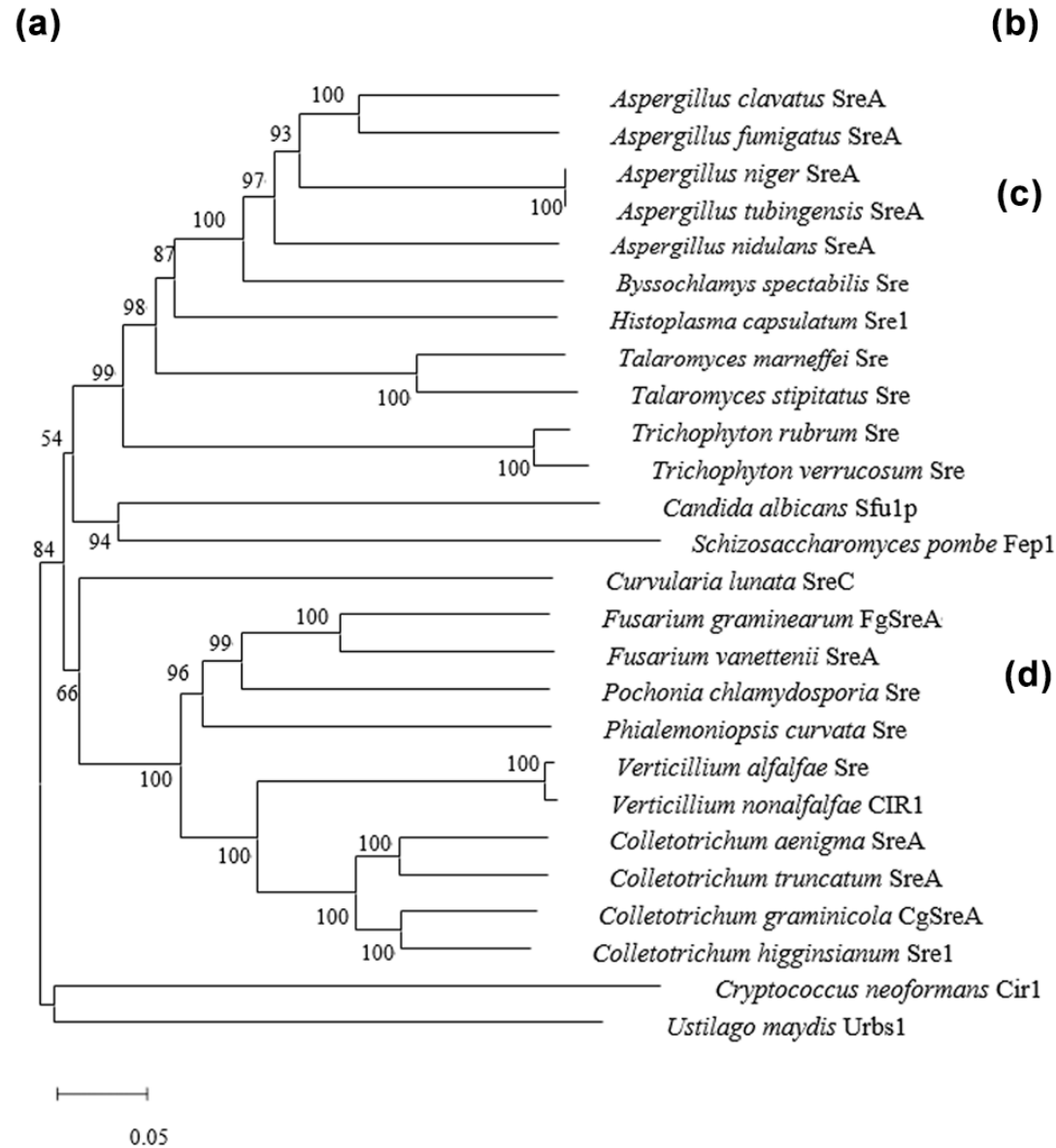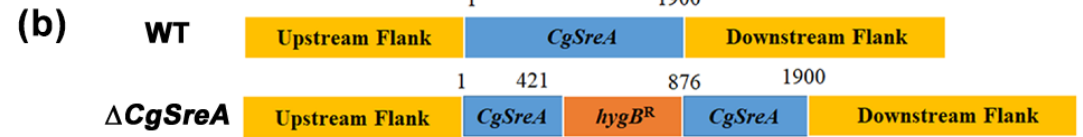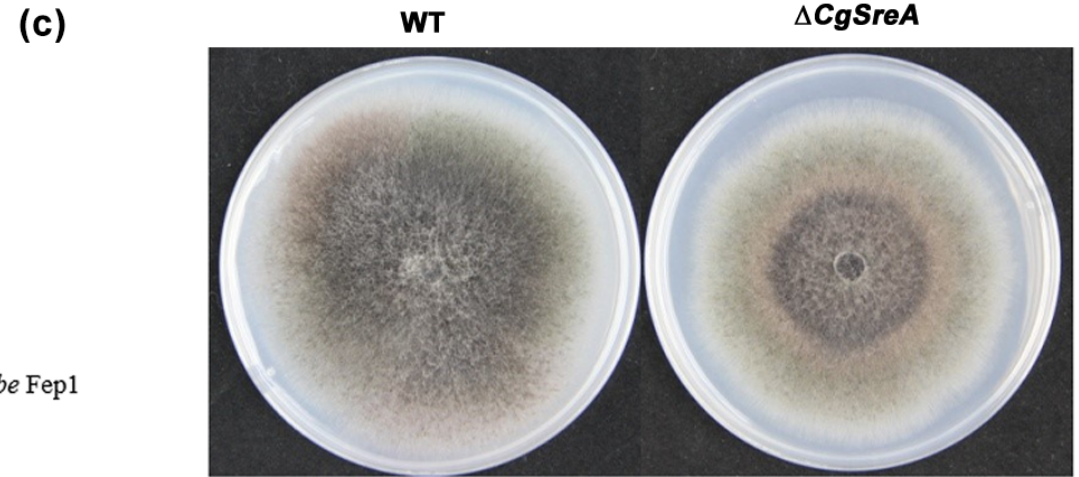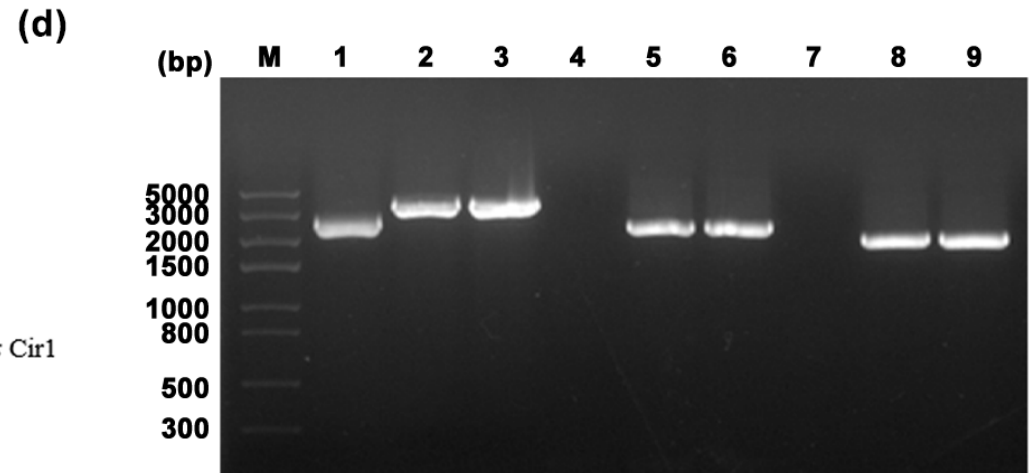

Supplement: Supplementary file 6 — FIGURE S6 Strategy of CgSreA deletion and verification of mutant strains. (a) Phylogenetic tree of CgSreA and its orthologues. All the amino acid sequences were aligned using the ClustalW program and the phylogenetic tree was constructed using MEGA 6 BETA program. All Sre protein sequences were downloaded from the NCBI database. (b) Gene replacement strategy for the deletion CgSreA. (c) Colony of wild type (WT) M1.001 and ΔCgSreA grown on minimal medium at 25°C for 7 days. (d) CgSreA gene knockout mutants Verification by PCR analysis. M, Trans 5K Marker. CX‐3, 1, 4, 7, 10. ΔCgSreA, 2, 3, 5, 6, 8, 9. [file MPP-25-e13444-s009.pdf]

(a)

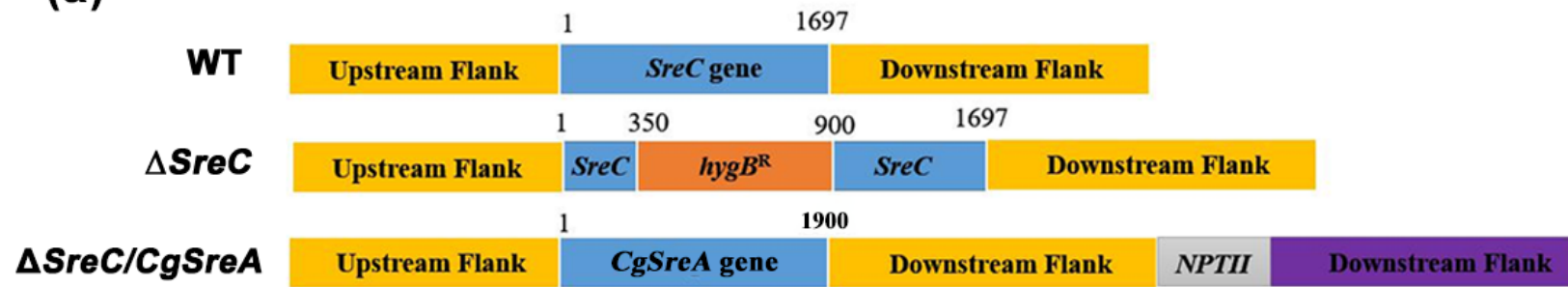

(b)

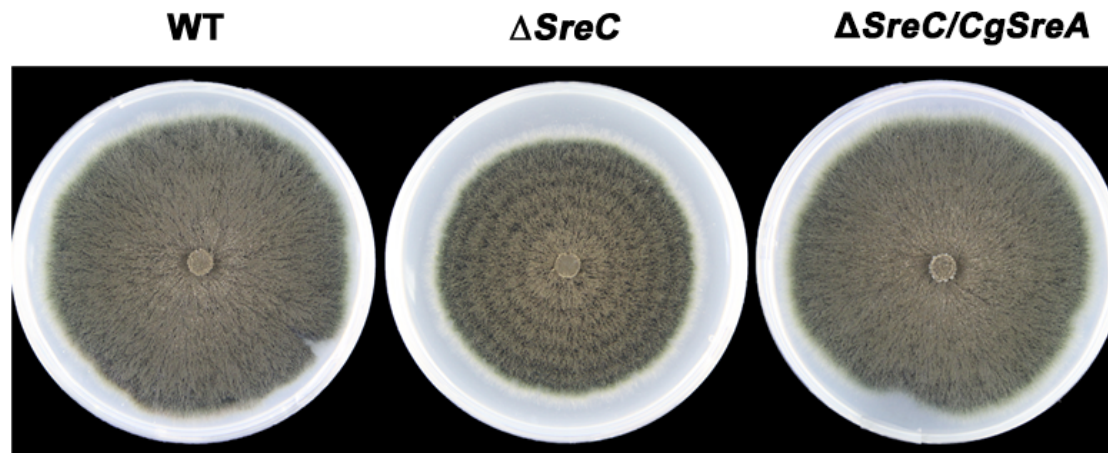

(c)

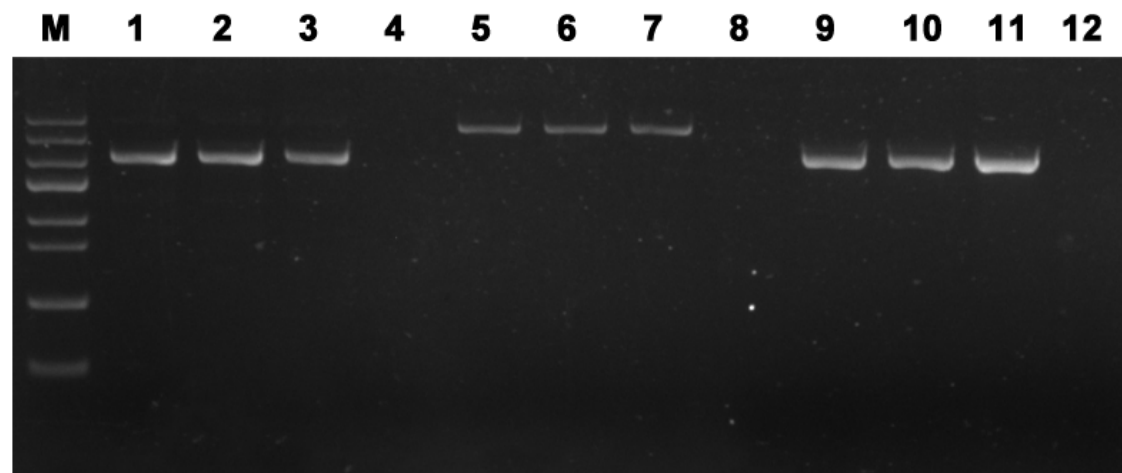

Supplement: Supplementary file 7 — FIGURE S7 Strategy of the CgSreA complement in the ΔSreC strain and verification ΔSreC/CgSreA strain. (a) Gene replacement strategy for ΔSreC/CgSreA. (b) Colony morphology of SreC gene knockout and ΔSreC/CgSreA complementary mutants. The wild‐type (WT) and mutant strains were grown in minimal medium for 7 days. (c) ΔSreC/CgSreA strain verification by PCR analysis. M, Trans 5K Marker. CX‐3, 4, 8, 12. ΔSreC/CgSreA, 1, 2, 3, 5, 6, 7, 9, 10, 11. [file MPP-25-e13444-s004.pdf]
