# Supplementary material for: SreC‐dependent adaption to host iron environments regulates the transition of trophic stages and developmental processes of Curvularia lunata
Source: Mol Plant Pathol. 2024 Mar 13;25(3):e13444. doi: 10.1111/mpp.13444 (PMC10938068; doi:10.1111/mpp.13444)
Supplement: Supplementary file 8 — FIGURE S8 CgSreA has similar function in Colletotrichum graminicola. (a) The C‐terminal zinc finger and cysteine‐rich central domains of CgSreA are required to interact with the promoters of genes in reductive iron assimilation (RIA) and siderophore‐mediated iron assimilation (SIA) pathways in yeast one‐hybrid hybridization system. Aureobasidin A (ABA) was added to the medium to inhibit the self‐activation of the CgSit1‐1 promoters. The positive control was CgSit‐1 promoter‐bait with AD‐CgSreA. The negative control was CgSit1‐1 promoter‐bait with AD. Concentration of ABA supplemented into synthetic dropout (SD) medium lacking leucine (L) (SD−L) was 200 ng/mL. (b) CgSreA interacted with CgGrx4 and CgFra2 in yeast two‐hybrid hybridization assay. Serial dilutions of the yeast cells were plated on SD medium lacking leucine (L), tryptophan (T), histidine (H), and adenine (A) (SD−L−T−H−A). The yeast strain containing pGBKT7‐53 and pGADT7 was used as a positive control, whereas that containing pGBKT7‐Lam and pGADT7 was used as a negative control. [file MPP-25-e13444-s007.pdf]

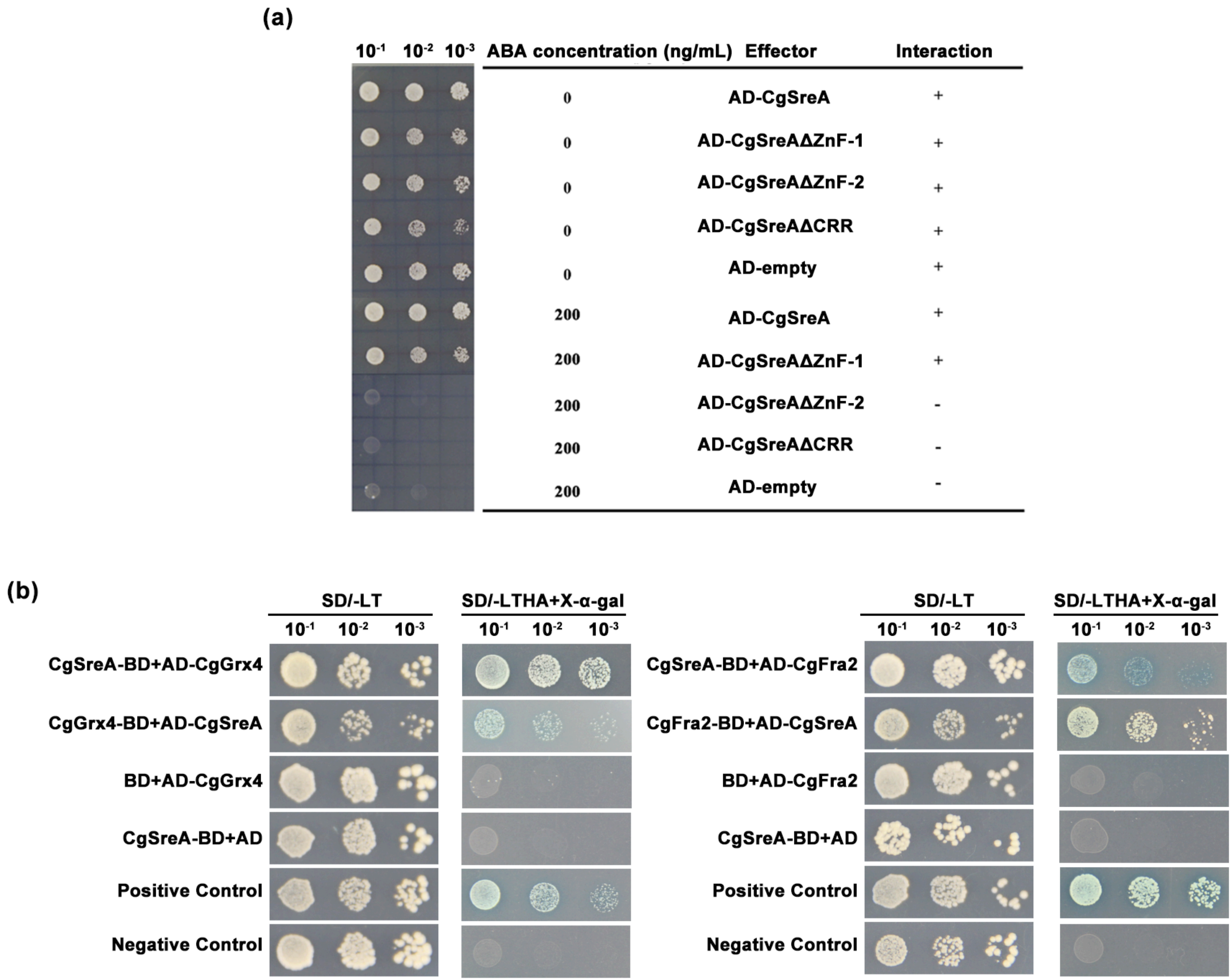
(a)
10^-1 10^-2 10^-3
ABA concentration (ng/mL)
Effector
Interaction
0 AD-CgSreA +
0 AD-CgSreAΔZnF-1 +
0 AD-CgSreAΔZnF-2 +
0 AD-CgSreAΔCRR +
0 AD-empty +
200 AD-CgSreA +
200 AD-CgSreAΔZnF-1 +
200 AD-CgSreAΔZnF-2 -
200 AD-CgSreAΔCRR -
200 AD-empty -
(b)
SD/-LT
SD/-LTHA+X-α-gal
10^-1 10^-2 10^-3
CgSreA-BD+AD-CgGrx4
CgGrx4-BD+AD-CgSreA
BD+AD-CgGrx4
CgSreA-BD+AD
Positive Control
Negative Control
CgSreA-BD+AD-CgFra2
CgFra2-BD+AD-CgSreA
BD+AD-CgFra2
CgSreA-BD+AD
Positive Control
Negative Control
