# Supplementary material for: SreC‐dependent adaption to host iron environments regulates the transition of trophic stages and developmental processes of Curvularia lunata
Source: Mol Plant Pathol. 2024 Mar 13;25(3):e13444. doi: 10.1111/mpp.13444 (PMC10938068; doi:10.1111/mpp.13444)
Supplement: Supplementary file 3 — FIGURE S3. SreC binds to the promoters of reductive iron assimilation (RIA) and siderophore‐mediated iron assimilation (SIA) pathways genes. (a) High‐affinity iron assimilation pathways for Curvularia lunata. RIA pathway occurred on the plasma membrane of C. lunata, extracellular Fe3+ was reduced to Fe2+ by ferric reductase ClFreB, Fe2+ by multicopper oxidase ClFet3p was oxidized to Fe3+, which was transported into the cell by iron permease ClFtr1. SIA pathway used L‐ornithine‐N‐5‐oxygenase ClSidA and non‐ribosomal peptide synthetase ClNps6 synthesizes siderophores and secreted them into extracellular to chelate iron to form siderophores‐ Fe3+, which was transported into the cell via a specific transporter ClSit1p and releases Fe3+. (b) Physical interaction of SreC with the promoters of RIA and SIA pathways genes in the yeast one‐hybrid hybridization system. Aureobasidin A (ABA) was supplemented into the medium to inhibit the self‐activation of the RIA and SIA pathway gene promoters. The negative control was genes promoter‐bait with AD. The concentration of ABA supplemented in synthetic dropout (SD) medium lacking leucine (L) (SD−L) was 200 ng/mL. [file MPP-25-e13444-s006.pdf]

(a)

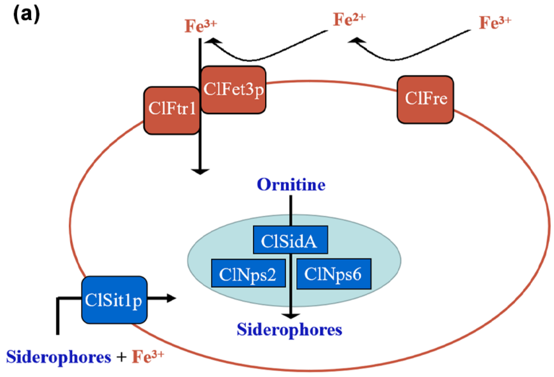

(b)

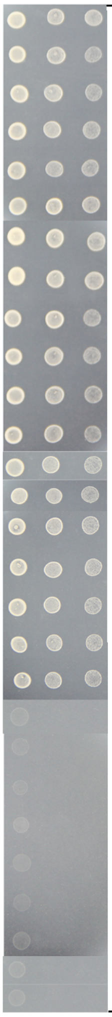

ABA concentration (ng/mL)	Reporter	Effector	Interaction
0	3×*ClFreB*-GATA	AD-SreC	+
0	3×*ClFtr1*-GATA	AD-SreC	+
0	3×*ClSidA*-GATA	AD-SreC	+
0	3×*ClNps2*-GATA	AD-SreC	+
0	3×*ClNps6*-GATA	AD-SreC	+
0	Mut-GATA	AD-SreC	+
0	Scramble-GATA	AD-SreC	+
0	3×*ClFreB*-GATA	AD-empty	+
0	3×*ClFtr1*-GATA	AD-empty	+
0	3×*ClSidA*-GATA	AD-empty	+
0	3×*ClNps2*-GATA	AD-empty	+
0	3×*ClNps6*-GATA	AD-empty	+
0	Mut-GATA	AD-empty	+
0	Scramble-GATA	AD-empty	+
200	3×*ClFreB*-GATA	AD-SreC	+
200	3×*ClFtr1*-GATA	AD-SreC	+
200	3×*ClSidA*-GATA	AD-SreC	+
200	3×*ClNps2*-GATA	AD-SreC	+
200	3×*ClNps6*-GATA	AD-SreC	+
200	Mut-GATA	AD-SreC	–
200	Scramble-GATA	AD-SreC	–
200	3×*ClFreB*-GATA	AD-empty	–
200	3×*ClFtr1*-GATA	AD-empty	–
200	3×*ClSidA*-GATA	AD-empty	–
200	3×*ClNps2*-GATA	AD-empty	–
200	3×*ClNps6*-GATA	AD-empty	–
200	Mut-GATA	AD-empty	–
200	Scramble-GATA	AD-empty	–
